# Analysis of a gene family for PDF-like peptides from Arabidopsis

**DOI:** 10.1038/s41598-021-98175-6

**Published:** 2021-09-23

**Authors:** Reza Omidvar, Nadine Vosseler, Amjad Abbas, Birgit Gutmann, Clemens Grünwald-Gruber, Friedrich Altmann, Shahid Siddique, Holger Bohlmann

**Affiliations:** 1 Division of Plant Protection, Department of Crop Sciences, Institute of Plant Protection, University of Natural Resources and Life Sciences Vienna, UFT Tulln, Konrad Lorenz Str. 24, 3430 Tulln, Austria; 2 Present Address: Institute of Biotechnology in Plant Production, Department of Agrobiotechnology, University of Natural Resources and Life Sciences, Vienna (BOKU), Tulln, Austria; 3 Present Address: Department of Plant Pathology, University of Agriculture, Faisalabad, 38040 Pakistan; 4 Present Address: RIVIERA Pharma and Cosmetics GmbH, Holzhackerstraße 1, Tulln, Austria; 5 Present Address: Department of Chemistry, University of Natural Resources and Life Sciences, Muthgasse 18, 1190 Vienna, Austria; 6 Present Address: Department of Entomology and Nematology, University of California Davis, Davis, CA 95616 USA

**Keywords:** Molecular biology, Plant sciences

## Abstract

Plant defensins are small, basic peptides that have a characteristic three-dimensional folding pattern which is stabilized by four disulfide bridges. We show here that Arabidopsis contains in addition to the proper plant defensins a group of 9 plant defensin-like (*PdfL*) genes. They are all expressed at low levels while GUS fusions of the promoters showed expression in most tissues with only minor differences. We produced two of the encoded peptides in *E. coli* and tested the antimicrobial activity in vitro. Both were highly active against fungi but had lower activity against bacteria. At higher concentrations hyperbranching and swollen tips, which are indicative of antimicrobial activity, were induced in *Fusarium graminearum* by both peptides. Overexpression lines for most *PdfL* genes were produced using the 35S CaMV promoter to study their possible in planta function. With the exception of *PdfL4.1* these lines had enhanced resistance against *F. oxysporum*. All PDFL peptides were also transiently expressed in *Nicotiana benthamiana* leaves with agroinfiltration using the pPZP3425 vector. In case of PDFL1.4 this resulted in complete death of the infiltrated tissues after 7 days. All other PDFLs resulted only in various degrees of small necrotic lesions. In conclusion, our results show that at least some of the *PdfL* genes could function in plant resistance.

## Introduction

A large variety of relatively small, basic, and often cysteine-rich polypeptides have been isolated from different organisms and shown to have antimicrobial activities in vitro^1–4^. Antimicrobial peptides (AMPs) are distributed ubiquitously in multicellular organisms. Plants have also been shown to contain a variety of different AMPs, including thionins^5,6^, plant defensins^7^, lipid transfer proteins and hevein-like peptides (reviewed by Garcia-Olmedo et al.^8^ and De Lucca et al.^9^. Cyclotides, found in the families *Violaceae*, *Rubiaceae*, and *Cucurbitaceae*, are cyclic peptides containing a cysteine knot^10^. It is thought that the molecular targets of the majority of the usually basic AMPs are acidic phospholipids in biomembranes^11^. However, at least for some AMPs, biomembranes may just be a barrier hindering access to their primary target inside the cell^12,13^.

Defensins are a large group of AMPs with a molecular weight in the range of 5 kDa which have been found in plants, animals, and fungi^7,14–18^. They share a common three-dimensional structure which is composed of one α-helix and a triple-stranded antiparallel β-sheet including both the cysteine-stabilised αβ (CSαβ) motif^15,19^ and the γ-core^4^. All plant defensins contain signal peptides and some defensins from *Solanaceae* are produced as preproproteins containing an acidic domain similar to thionins^20,21^. Many plant defensins are constitutively expressed in seeds and have been isolated from these organs (for instance Terras et al.^22^ and Rogozhin et al.^23^ and some are inducible through pathogen infection (for instance Chiang and Hadwiger^24^). Defensins have repeatedly been shown to have antimicrobial activity in vitro^25,26^ and for some anti-insect activities were reported^27^. In addition, especially those plant defensins, that were originally called γ-thionins, inhibit α-amylase activity^28^ and protein synthesis^29^.

Arabidopsis has 13 *Pdf* genes that were divided into two groups according to Thomma et al.^7^. Three genes of group 1 (*Pdf1.2a*, *Pdf1.2b*, and *Pdf1.2c*) are very closely related and encode the same defensin peptide. *Pdf1.2* is generally regarded as a marker for pathogen specific induction through the ethylene and jasmonic acid pathways^30^. Other Arabidopsis *Pdf* genes are constitutively expressed in certain plant tissues^31^. Group 1 *Pdf* genes are induced in the non-host response of Arabidopsis to the barley powdery mildew fungus^32^. Some Arabidopsis PDF peptides have been shown to have in vitro antimicrobial activity, for instance PDF1.1 and PDF1.3^33^. Overexpression of PDF1.1 resulted in enhanced resistance of Arabidopsis plants against *Cercospora beticola*^34^. PDFs might also exert their defensive function by a metal binding activity. It was recently reported that PDF1.1 protects the plant against *Pectobacterium carotovorum* via iron capture^35^. Iron is an essential element for pathogenic microorganisms. PDF2.5 and PDF2.6 have been reported to chelate cadmium^36,37^.

While in the past AMPs have been generally discovered through purification from natural sources, guided by their antimicrobial activity (for instance Broekaert et al.^38^), it is now possible to identify the corresponding genes/cDNAs in various EST or genomic databases^39–43^. In an analysis of the Arabidopsis genome^42^, 317 *DEFL* (defensin-like) genes coding for small cysteine-rich peptides (CRPs) were discovered. Most of these genes were previously not annotated and apparently lacking from the Affymetrix Arabidopsis GeneChip. These genes were grouped into families from CRP0000 to CRP1520. CRP0000, CRP0030, CRP0090 and CRP0100 include the original 13 plant defensin genes.

While the DEFL peptides could have antimicrobial activity, at least some of these newly discovered putative DEFL peptides might have other functions than in plant defence against pathogens. Several DEFL genes are involved in reproduction, for instance as pollen tube attractants^44^. Plant defensins have been shown to be involved in conferring zinc resistance in the zinc hyper-accumulating plant *Arabidopsis halleri*^45^. *AhPDF1.1* was found to accumulate in intracellular compartments instead of being secreted^46^.

In mammals it has been demonstrated that peptides related to AMPs can modulate innate immunity^47,48^ and also plants contain peptides that are not directly antimicrobial but are modulators of defence responses. Systemin is a peptide discovered in tomato which is involved in the wound response^49^. A functionally related peptide has recently been discovered in Arabidopsis^50,51^. In addition, plants contain a variety of other peptides that function in signalling (reviewed by Murphy et al.^52^).

Orphan genes lack homologues in other lineages. They are unique to a narrow taxon, usually a species^53^. Studies of Arabidopsis have reported 958^54^, 1324^55^, 1430^56^, and 1084 orphan genes^53^, respectively. The differences in number could be due to the genome datasets that were used and the methods to identify them. Anyway, a large number of genes in Arabidopsis seem to be orphans. The average exon length of orphans is usually rather small with 50 amino acids and many orphan genes play a role in resistance against abiotic and biotic stress^53^. One example is the Arabidopsis *EWR1* gene which was found to be involved in resistance to vascular wilt pathogens^57^.

We have here analysed the 9 genes in group CRP0240. We call them *PdfL* (plant defensin-like). We have analysed the expression using RT-PCR and promoter::GUS lines. Overexpression showed enhanced resistance against *Fusarium oxysporum* f sp *matthiolae* for several genes. We also expressed 2 of the peptides in *E. coli* and found strong antimicrobial activity against fungi in vitro.

## Materials and methods

### *E. coli* strains and pathogens

Vectors and *E. coli* strains are listed in Table S1. We used the *E. coli* DH10B strain for cloning. For protein expression, the pETtrx1a vector^58^ was transformed into the *E. coli* SHuffle strain C3030^59^. For in vitro tests, we used the bacteria *E. coli* DH5alpha and *Pseudomonas syringae* pv *tomato* DC3000 (*Pst* DC3000) and the fungi *Fusarium oxysporum* f sp *matthiolae* (Centraalbureau voor Schimmelcultures (CBS), Baarn-Delft, Netherlands), *F. graminearum* (provided by Marc Lemmens), and *B. cinerea* (isolated in our lab). In planta infection tests were performed on *Arabidopsis thaliana* Columbia plants using *F. oxysporum.*

### Cloning of binary vectors

The vector pPZP3425^60^ was used for promoter analysis and overexpression studies. The vector contains a double enhanced 35S promoter and TMV omega element driving an intron containing GUS gene. Promoter regions were amplified by PCR using Arabidopsis genomic DNA as template. PCR primers (Table S2) included restriction sites for KpnI or EcoRI and NcoI. The PCR fragments were digested with KpnI or EcoRI and NcoI and ligated into the vector fragment of pPZP3425 previously digested with the same enzymes. The 35S promoter was thus replaced by the different *PdfL* promoters. For cloning of the overexpression vectors, the coding sequences were amplified from genomic DNA by PCR using primers containing the restrictions sites for NcoI and BamHI (Table S3). The amplified PCR products were digested with NcoI and BamHI and ligated into the vector pPZP3425 digested with the same enzymes. All constructs were confirmed by sequencing.

### Plant material and growth conditions

Arabidopsis seeds (ecotype Columbia) were surface sterilized in 6% (w/v) sodium hypochlorite for 20 min and then washed three times with sterile water. Plants were grown under sterile conditions on Murashige and Skoog (MS) medium^61^. Plants were grown on soil for seed production or on MS medium in 5 cm Petri dishes for resistance tests against *F. oxysporum* in a growth chamber at 25 °C in a 16 h light and 8 h dark cycle.

Collection of plant material, must comply with relevant institutional, national, and international guidelines and legislation.

### Arabidopsis transformation

Binary vectors were transformed into *Agrobacterium tumefaciens* GV3101 using the freeze/thaw method^62^ and then transferred into *Arabidopsis* plants by a modified floral dip method^63^. Transformed seedlings were selected on MS medium with 50 μg/ml kanamycin and 250 μg/ml timentin at 22 °C with a photoperiod of 16 h light and 8 h dark. Kanamycin-resistant seedlings were transferred to soil for seed production.

For each promoter::GUS construct, 10–12 independent transgenic plants were generated and tested for GUS activity. Two representative lines were then grown further to produce homozygous lines. For overexpression lines, 12 or more independent transgenic T2 lines were generated and applied to RT-PCR to select the best expressing lines. These were then made homozygous to be used in resistance tests.

### GUS reporter analysis

Histochemical detection of GUS activity was performed according to Jefferson^64^. Plant tissues were incubated in X-gluc (Biomol, Hamburg, Germany) solution consisting of 0.1 M sodium phosphate buffer pH 7.0, 0.1% Triton-X 100, 0.5 mM K_3_[Fe(CN)_6_], 0.5 mM K_4_[Fe(CN)_6_] and 10 mM Na_2_EDTA at 37ºC overnight. After staining, chlorophyll was removed from photosynthetic tissues with 70% (v/v) ethanol.

### Semi-quantitative RT-PCR

Total RNA was purified from all independent plant lines using the “NucleoSpin^®^ RNA Plant” from Macherey-Nagel. 2 μL of eluted RNA were used for photometric measurement of RNA concentration (NanoDrop 2000, Thermo Scientific). Semi-quantitative RT-PCR was done with a “One-step Master Mix RT-PCR Kit” (Affymetrix) according to the manufacturer's instructions using the primers described in Table S4.

### Protein expression and purification

Sequences of mature PDFL1.1 (*At1g64195*) and PDFL2.1 (*At1g35537*) were cloned (primers and size of PCR products are shown in Table S5 and S6, respectively) as a fusion protein with thioredoxin into a derivative of the pETtrx_1a vector^58^ and expressed in the Shuffle strain C3030 of *E. coli*^59^. The fusion proteins were isolated by metal chelating chromatography and cleaved with TEV protease to release the PDFL1.1 and PDFL2.1 as previously described for thionin proproteins^65^. PDFL1.1 and PDFL2.1 were further purified by HPLC using a reversed phase SOURCE 15RPC ST 4.6/100 mm column and characterized by mass spectrometry.

PDFL1.1 was analysed using a LC–ESI–MS system (Bruker maXis 4G QTOF). The sample was loaded on a BioBasic C18 column (BioBasic-18, 150 × 0.32 mm, 5 µm, Thermo Scientific) using 80 mM ammonium formate buffer as the aqueous solvent. A gradient from 5% B (B: 80% acetonitrile) to 100% B in 25 min was applied, at a flow rate of 6 µL/min, directly. The QTOF was equipped with the standard ESI source in positive ion, DDA mode (= switching to MSMS mode for eluting peaks). MS-scans were recorded (range 150–2200 Da) and peptide was identified by the exact mass. MS/MS Scans were recorded, to confirm the sequence (data not shown). Instrument calibration was performed using ESI calibration mixture (Agilent).

PDFL2.1 was characterized with TOF-MS which was conducted with a QSTAR XL Quadrupole TOF MS instrument from AB Sciex. The peptide was diluted in a 1:1 ratio in a solution of 50% acetonitrile containing 0.1% formic acid and directly injected into the mass spectrometer at a rate of 5 μl/min. The data were deconvoluted using the Analyst Software package from AB Sciex.

### In vitro antimicrobial assays

Antimicrobial activity of the purified peptides was tested in 96-well microtiter plates against the bacteria *E. coli* DH5alpha and *Pst DC3000* and the fungi *Fusarium oxysporum* f.sp. *matthiolae*, *Fusarium graminearum*, and *Botrytis cinerea*. Assays were done in sterile 96-well flat-bottomed microtiter plate (Greiner Bio-One) pre-loaded with 25 μl of different concentrations of filter-sterilized (0.22 μm syringe filter, Roth) PDFL1.1, PDFL2.1 or ddH_2_O. In antibacterial assays 75 µl of the bacterial cell culture with an OD_600_ of 0.05 in a minimal medium (K_2_HPO_4_ 2.5 mM, MgSO_4_ 50 μM, CaCl_2_ 50 μM, FeSO_4_ 5 μM, CoCl_2_ 0.1 μM, CuSO_4_ 0.1 μM, Na_2_MoO_4_ 2 μM, H_3_BO_4_ 0.5 μM, KI 0.1 μM, ZnSO_4_ 0.5 μM, MnSO_4_ 0.1 μM, glucose 10 g, asparagine 1 g, methionine 20 mg, myo-inositol 2 mg, biotin 0.2 mg, thiamine-HCl 1 mg, pyridoxine–HCl 0.2 mg in 1 L ddH_2_O) was added to each well. For antifungal assays a fungal spore suspension with a concentration of 2 × 10^4^ spores per ml in 1/4 strength potato dextrose broth was added to the wells. The plates were incubated in a FLUOstar Omega plate reader (BMG LabTech) at 37 °C, 30 °C and room temperature (RT), respectively for *E. coli*, *P. syringae* and all tested fungi. For bacterial strains, OD_600_ was measured every 30 min with 5 min shaking of the plate at 500 rpm before each measurement within a period of 24 h while in antifungal assays, OD_600_ was measured at two time points, after 30 min and 24 h. The IC_50_ value was determined as the concentration of the peptide required for 50% growth inhibition. All assays were done in triplicate.

### Resistance test against *F. oxysporum*

For resistance test against *F. oxysporum* f. sp. *matthiolae*, Arabidopsis seeds from *PdfL* overexpression lines were surface sterilized in 6% (w/v) sodium hypochlorite for 20 min and then washed three times with sterile water. Plants were grown under sterile conditions on MS medium in 5 cm Petri dishes under long day conditions at 25 °C for 12 days. Then, under sterile conditions, plants in each Petri dish were sprayed with 1.6 ml of a spore suspension of the fungus with a concentration of 10^5^ spores per ml. For each line, 4 Petri dishes were infected which were then placed back in the growth chamber under the same conditions (only the first 24 h in darkness). At 5 dpi, twenty seedlings were randomly harvested from each Petri dish and stained by trypan blue as described by Keogh et al.^66^. Destaining was performed in chloral hydrate solution (125 g chloral hydrate in 50 ml water) overnight and seedlings were then stored in 50% glycerol. Microscopy was done using an Olympus BX53 microscope. Infected seedlings were assigned to four classes (Figure S1) based on the fungal growth on a cotyledon: no visible infection (class 0), 1–20 hyphae (class 1); 20–100 hyphae (class 2); densely covered cotyledon (class 3). A disease index (DI) was calculated as described by Epple et al.^67^. Forty cotyledons from each Petri dish were evaluated for calculation of the disease index and the mean value from 4 Petri dishes was considered as the disease index for each line and finally compared to the ecotype Columbia.

### Transient expression in *Nicotiana benthamiana*

The pPZP3425 vectors containing the overexpression constructs of all PdfL genes were transformed into *Agrobacterium tumefaciens* strain GV3101 using the freeze–thaw method^62^. Agrobacteria were grown overnight in YEB liquid medium with appropriate antibiotics (25 μg/ml gentamicin and 35 µg/ml rifampicin for Agrobacteria and 50 μg/ml kanamycin for pPZP3425 vector)^68^ to an OD_600_ of 1 in an incubator/shaker at 28 °C. Bacteria were then harvested by centrifugation at 5000 rpm for 7 min in a table top centrifuge at RT, resuspended in an infiltration medium (10 mM MES pH 5.6; 10 mM MgCl_2_ and 100 μM acetosyringone) to an OD_600_ of 1 and incubated for 2 h at RT. Before infiltration, the bacterial suspension was mixed with an equal volume of a bacterial suspension harbouring pBin61-P19^69^ to co-introduce the T-DNA for both the gene of interest and the RNA-silencing suppressor gene into the cells. The mixture of Agrobacterium suspensions was then infiltrated using a 1-ml syringe without needle into the abaxial side of second, third and fourth leaves of 4 weeks old *N. benthamiana* plants grown in a growth chamber at 25 ± 2 °C temperature, 16-h photoperiod and ~ 65% humidity. Infiltrated plants were placed back into the same growth chamber and checked for a HR reaction after 3 days.

### Bioinformatic analysis

MW and pI were computed at https://web.expasy.org/compute_pi/. Clustal Omega (https://www.ebi.ac.uk/Tools/msa/clustalo/) was used for multiple sequence alignments^70^. An unrooted tree for the mature peptides was calculated using W-IQ-TREE (http://iqtree.cibiv.univie.ac.at). Input alignments of the mature domains of all PDFLs in Clustal format was submitted to the W-IQ-TREE online phylogenetic tool and the created tree was downloaded and analysed^71^. The putative signal peptide was predicted using the SignalP 4.0 Server (http://www.cbs.dtu.dk/services/SignalP/). Sequence logos were created at http://weblogo.threeplusone.com/create.cgi^72^. 3D structure prediction was done at PHYRE^73^. Genomic data were retrieved from PLAZA (https://bioinformatics.psb.ugent.be/plaza/)^74^.

## Results

**Figure 1:**
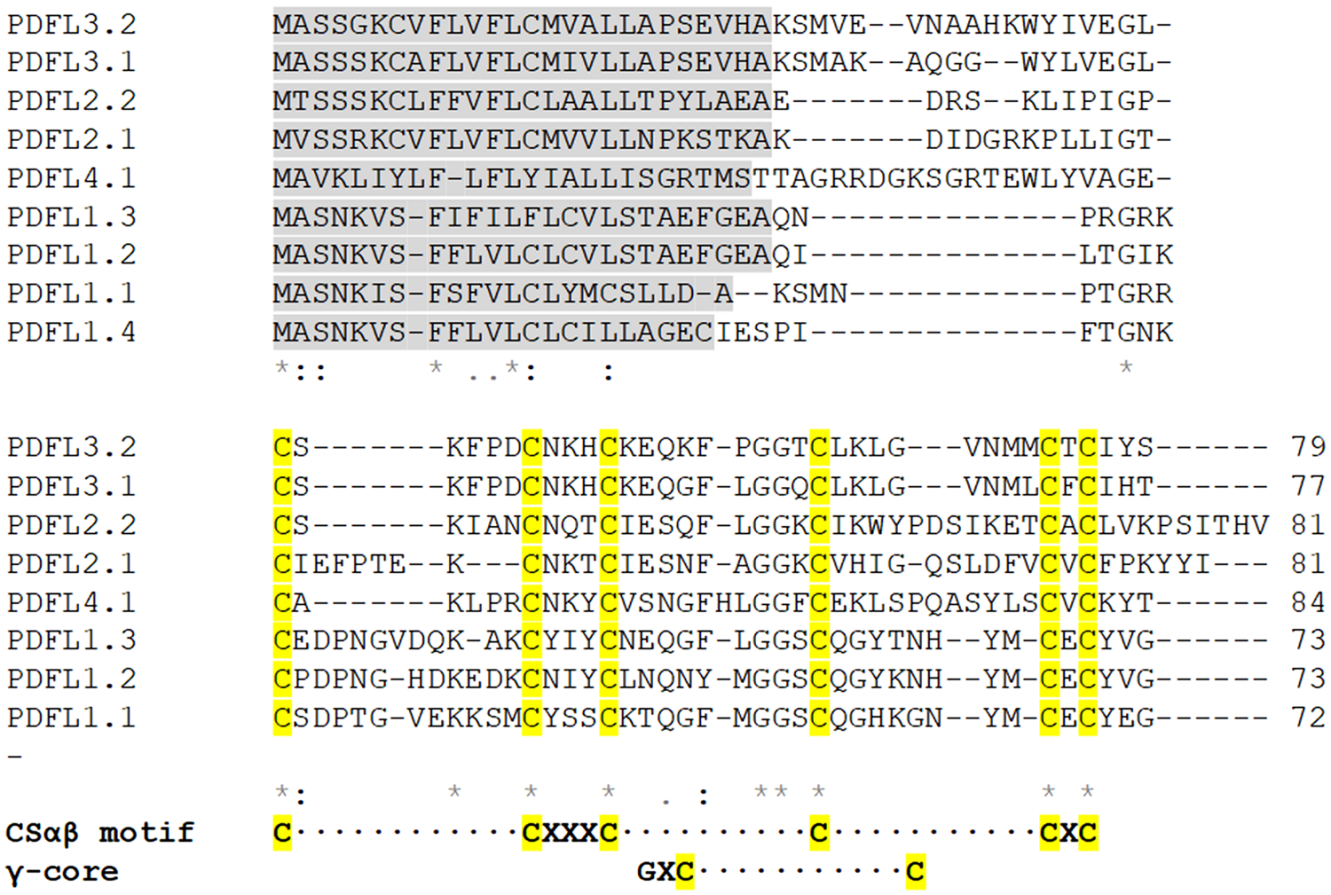
Multiple sequence alignment of all 9 PDFL precursor peptides. Alignment with CLUSTAL W (1.82) (http://www.ebi.ac.uk/Tools/clustalw/index.html). Symbols below the alignment are as follows: *indicates perfect alignment, : indicates strong similarity of the aligned amino acids and indicates weak similarity of the aligned amino acids. The putative signal peptide (prediction according to SignalP 4.0 Server http://www.cbs.dtu.dk/services/SignalP/) is shaded and cysteines are yellow. Additional gaps have been introduced manually for better alignment. The CSαβ motif and the γ-core are shown below the sequences.

**Figure 2:**
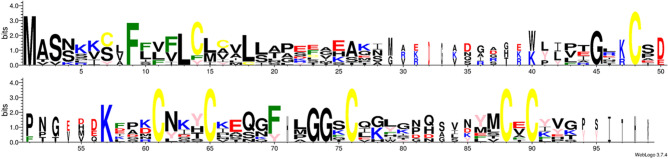
Sequence logo of PDFL precursor proteins. Signal peptide boxed in grey. Cysteine yellow, basic amino acids blue, acidic amino acids red, tyrosine pink, phenylalanine green.

**Figure 3:**
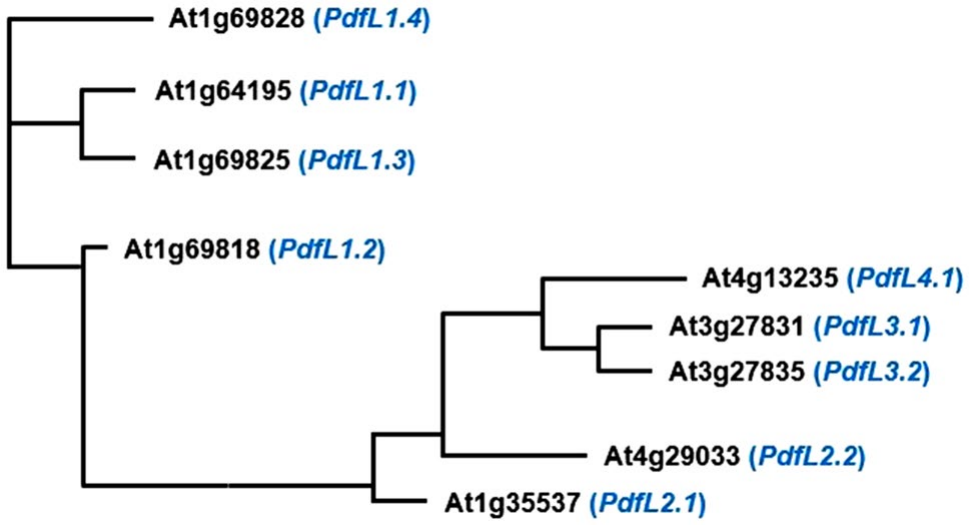
Phylogenetic tree of all 9 PDFL peptides. Alignment of the mature peptides was done with CLUSTAL Omega (http://www.ebi.ac.uk/Tools/clustalw/index.html). Tree was calculated at http://iqtree.cibiv.univie.ac.at.

The CRP0240 group contains 9 genes. We have named them *PdfL* (plant defensin-like). All genes contain a small intron within the coding sequence. According to SignalP all genes encode signal peptides (Fig. 1). All signal peptides contain a phenylalanine residue at position 6 and most of them contain 2 cysteines. Only the PDFL4.1 signal peptide does not contain a cysteine. The mature peptides all contain 6 cysteine residues, the CSαβ motif^15^ and the γ-core^4^ specific for antimicrobial peptides (Fig. 1). Besides the cysteine residues only few amino acids are conserved. These include 2 glycines at the beginning of the γ-core (the second one is part of the γ-core) and one glycine 2 or 3 amino acids downstream from the first cysteine (Figs. 1, 2). We have grouped the *PdfL* genes into 4 subfamilies (Table 1) as shown in the phylogenetic tree (Fig. 3). This corresponds with the PLAZA gene families for this group, except that we have divided the PLAZA gene family HOM04D008815 into 2 subfamilies. The size of the mature PDFL peptides is around 55 amino acids with pIs between 4.6 and 9 (Table 1). The acidic peptides are all found in subgroup 1, together with one basic peptide (PDFL1.1).

**Table 1:** MW and pIs of PDFL mature peptides.

Name	PLAZA	New Gene Symbols	pI	MW	1	2
*At1g64195*	HOM04D011286	*PdfL1.1*	8.62	5474	X	X
*At1g69818*		*PdfL1.2*	6.02	5450	X	X
*At1g69825*		*PdfL1.3*	6.75	5429	X	
*At1g69828*		*PdfL1.4*	4.62	5511	X	X
*At1g35537*	HOM04D008815	*PdfL2.1*	7.63	6144	X	X
*At4g29033*		*PdfL2.2*	8.01	6068	X	X
*At3g27831*		*PdfL3.1*	8.30	5624	(X)	
*At3g27835*		*PdfL3.2*	8.30	5972	X	
*At4g13235*	HOM04D013081	*PdfL4.1*	9.04	6613		

1, orphans according to Lin et al.^55^; 2, orphans according to Donoghue et al.^54^.

Most of the *PdfL* genes, except *PdfL4.1*, were classified as orphan genes by Lin et al.^55^ and Donoghue et al.^54^. However, as the number of sequenced genomes has increased, it has become clear, that the *PdfL* genes are not orphans but the group has been expanded in the *Brassicaceae* plant family according to data at PLAZA (Figure S2). There are several homologous genes in the *Brassicacea* species *A. lyrata*, *Brassica rapa*, *B. oleracea*, *Capsella rubella* and *Thellungiella parvula*. In addition, there is one homologous gene in *Tarenaya hassleriana* (family *Cleomaceae*, order *Brassicales*, clade *Rosids*) and one in *Gossypium raimondii* (family *Malvaceae*, order *Malvales*, clade *Rosids*).

We submitted the peptide sequences to a 3D structure prediction at PHYRE^73^. The structure of PDFL2.1 had been determined before^75^ and for PDFL2.2 the prediction was for a very similar structure with one α-helix and three β-sheets (Figure S3). For PDFL3.1 and PDFL3.2 the template was also PDFL2.1 leading to models with two α-helices and two β-sheets and one α-helix and three β-sheets, respectively. The structure of PDFL4.1 contained one α-helix and three β-sheets and the template was also PDFL2.1 but the prediction had only a low confidence level. The models for PDFL1.1, PDFL1.2, PDFL1.3 and PDFL1.4 had one α-helix and two β-sheets and were modelled mainly against a plant defensin from *Vigna radiata*^76^. Thus, according to these models, the structures of most PDFL peptides would resemble the structure of plant defensins.

### Expression of *PdfL* genes in different plant tissues

**Figure 4:**
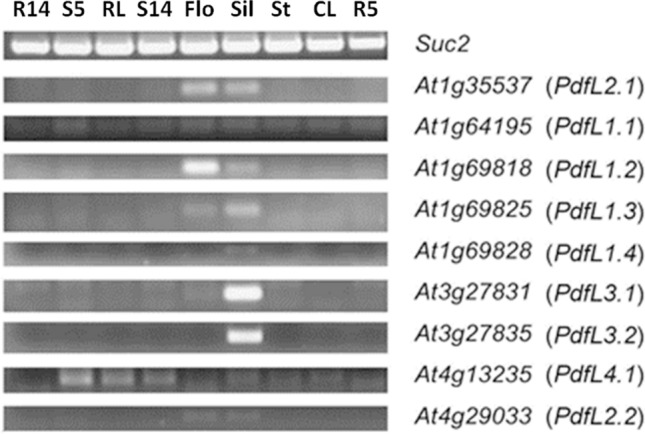
RT-PCR for all CRP0240 genes in different Arabidopsis tissues. *R14* roots 14 days on MS medium, *S5* seedlings 5 days MS, *RL* rosette leaves 5 weeks, *S14* seedlings 14 days MS, *Flo* flowers, *Sil* siliques, *St* stems, *CL* cauline leaves, *R5* roots 5 days MS. Total RNA was transcribed using oligo-dT and superscriptIII reverse transcriptase and amplified using gene-specific primers placed on both sides of the intron. Specificity of the reaction was tested with genomic DNA [shown on full-length gels in Figure S4)].

Expression analysis using RT-PCR showed that all genes are expressed at a very low level (Fig. 4, full-length gels shown in Figure S4). The highest expression was found in flowers (*PdfL2.1* and *PdfL1.2*) or siliques (*PdfL3.1* and *PdfL3.2*) for some of the genes. GeneChip data are only available for *PdfL4.1* confirming a low expression level (data not shown). A custom microarray for all *DEFL* genes^77^ also shows a low expression of all *PdfL* genes (Table S7) in different organs. In addition, using the DEFL microarray, the authors did not find induction of the *PdfL* genes after infection by different pathogens (Tables S8 and S9).

**Figure 5:**
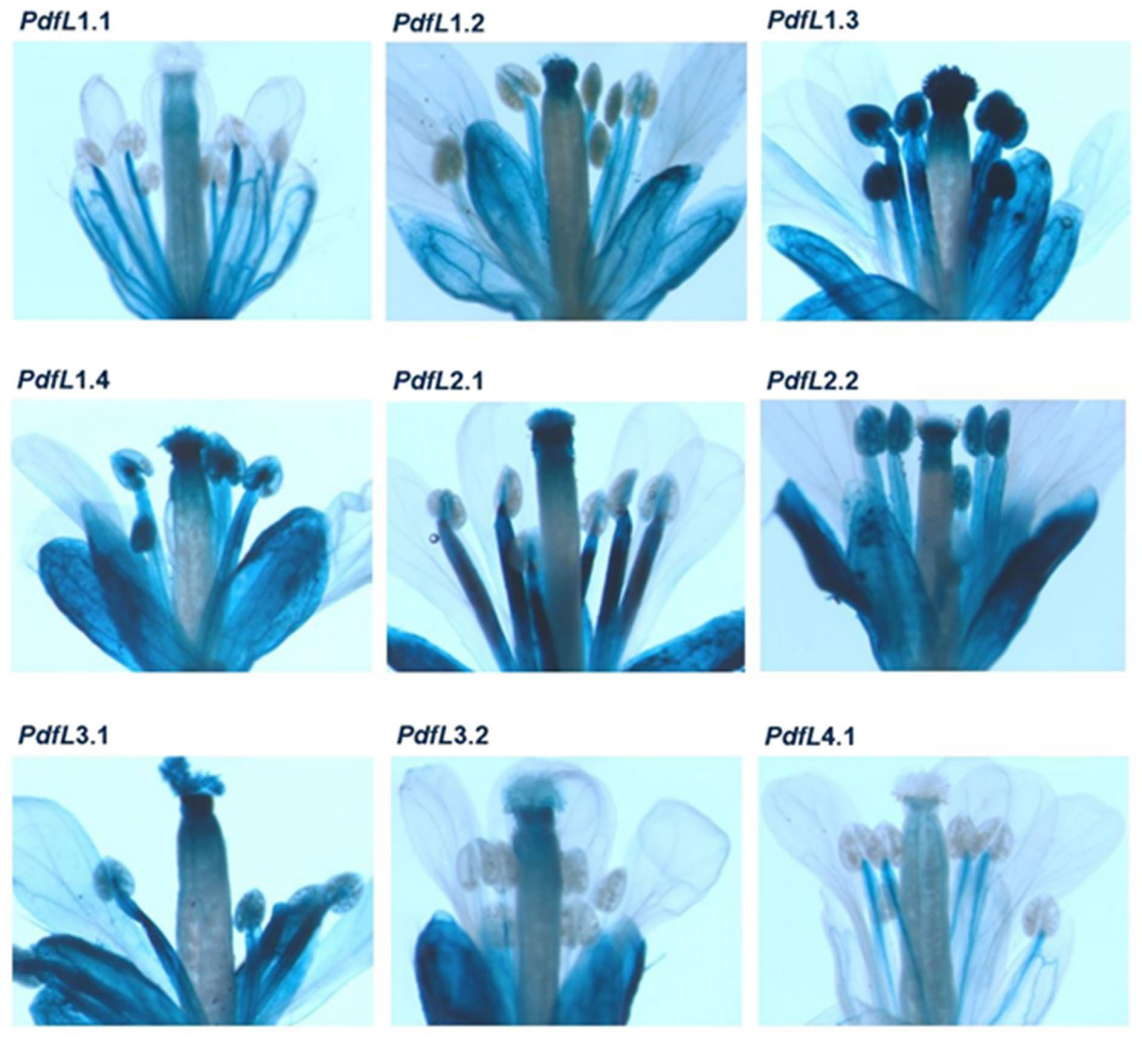
Expression of *PdfL*s in flowers. GUS staining of promoter::GUS lines.

To further study the tissue-specific expression, we produced promoter::GUS lines for all genes. GUS staining (Table 2 and Figures S5–S13) showed promoter activity throughout the plant with some exceptions. There was no staining in root tips and petals in any GUS line. In stigmas no GUS staining was found in the lines with *PdfL1.1* and *PdfL4.1* promoters. There was no GUS staining in most GUS lines for hypocotyls, ovaries and anthers. Furthermore, staining was restricted to parts of some reproductive organs and sepals for some lines (Table 2; Fig. 5).

**Table 2:**
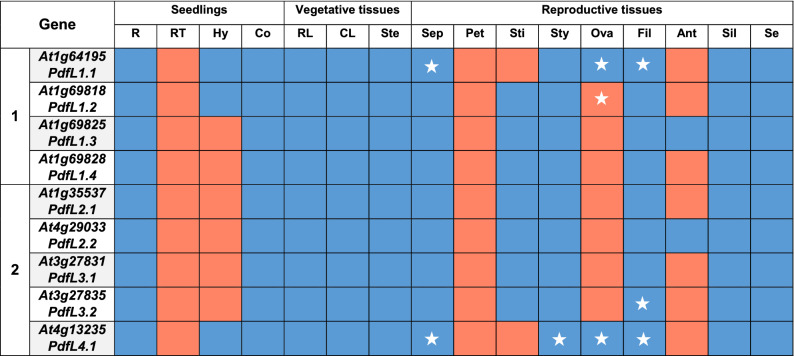
Comparison of the activity of the promoters of 9 *PdfL* genes in different plant tissues.

Blue indicates expression and red indicates no expression. White stars indicate a different expression pattern than others: *PdfL1.1* and *PdfL4.1* are expressed only in the veins of sepals. The promoter of *PdfL4.1* is only active in the vascular tissue of the style. The *PdfL1.1* promoter has weak activity throughout the ovary and also in the vascular tissues while the promoter of *PdfL4.1* is active in the vascular tissue of the ovary. The promoter of *PdfL1.2* has no activity in the ovary while activity of the promoters of other *PdfLs* is restricted activity to the upper part of the ovary connected to the style. Activity of the promoters of *PdfL1.1* and *PdfL4.1* was stronger in the vascular tissue of the filament than the other tissues and the activity of the *PdfL3.2* promoter was restricted to the vascular tissue of the filament. *R* root, *RT* root tip, *Hy* hypocotyl, *Co* cotyledon, *RL* rosette leaves, *CL* cauline leaves, *Ste* stem, *Sep* sepal, *Pet* petal, *Sti* stigma, *Sty* style, *Ova* ovary, *Fil* filament, *Ant* anther, *Sil* siliques, *Se* seed.

### In vitro antimicrobial activity of PDFL1.1 and PDFL2.1 peptides

**Figure 6:**
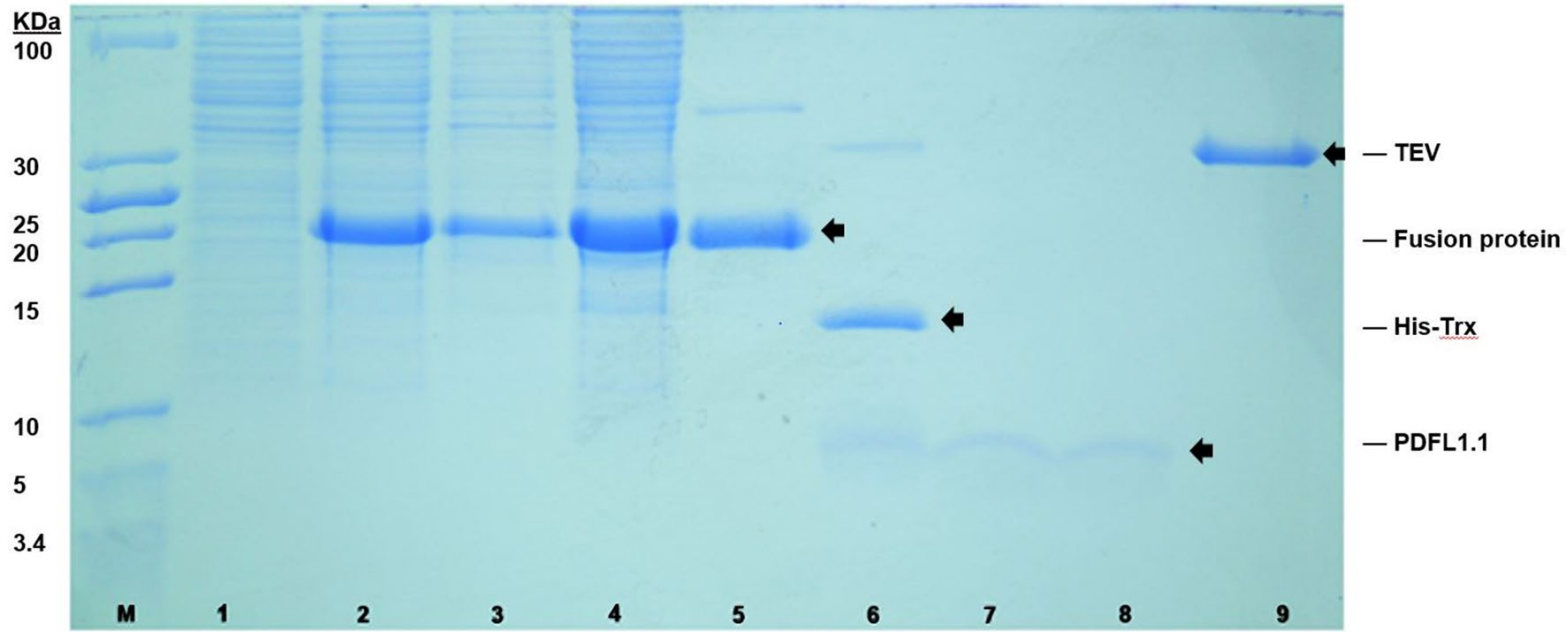
Purification of PDFL1.1. 1: Uninduced crude fraction, 2: Induced crude fraction, 3: Insoluble fraction, 4: Total soluble protein, 5: Elution after purification, 6: Digestion of the purified fusion protein with TEV protease. 7: Second His-tag purification after TEV digestion, 8: Purified PDFL1.1 after chromatography, 9: TEV protease. M: Low range protein ladder.

**Figure 7:**
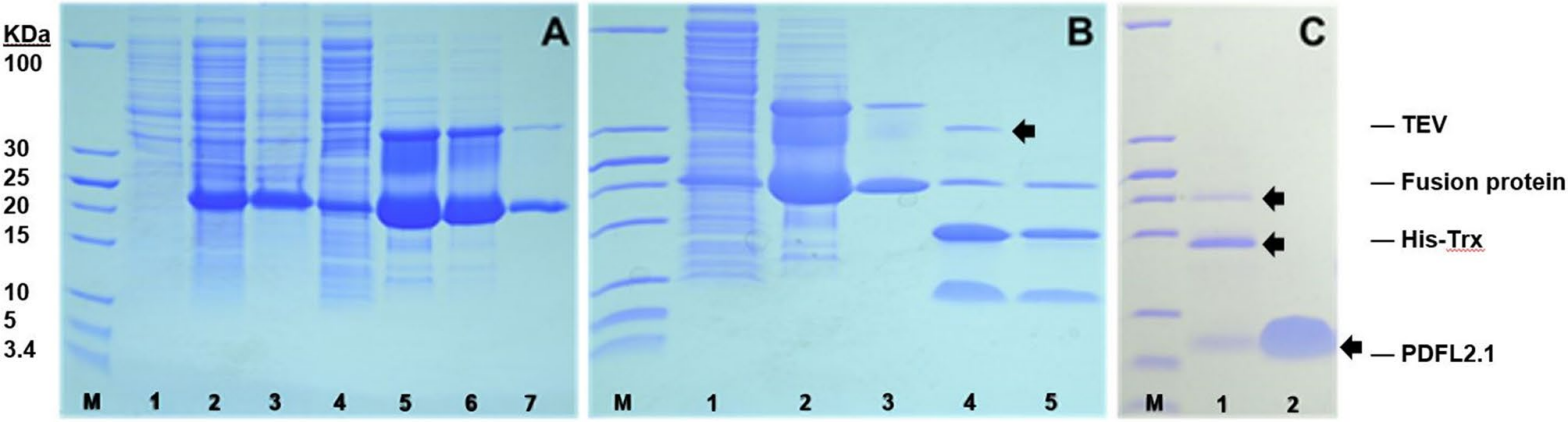
Purification of PDFL2.1. (**A**) His-tag purification of fusion protein. 1: Uninduced crude protein extract, 2: Induced crude protein extract, 3: Total soluble protein, 4: Insoluble fraction, 5–7: First, second and third elution after purification. (**B**) Digestion of the purified fusion protein with TEV protease. 1: Total soluble protein, 2: Eluted protein after His-tag purification, 3: Protein after acetone precipitation and dissolving in TEV reaction buffer, 4: TEV digested fusion protein, 5: Second His-tag purification after TEV digestion. (**C**) Final purification of PDFL2.1 using reverse phase chromatography. 1: TEV digested fusion protein, 2: Purified PDFL2.1 after chromatography. M: Low range protein ladder. Full-length gels are shown as Figure S14.

**Figure 8:**
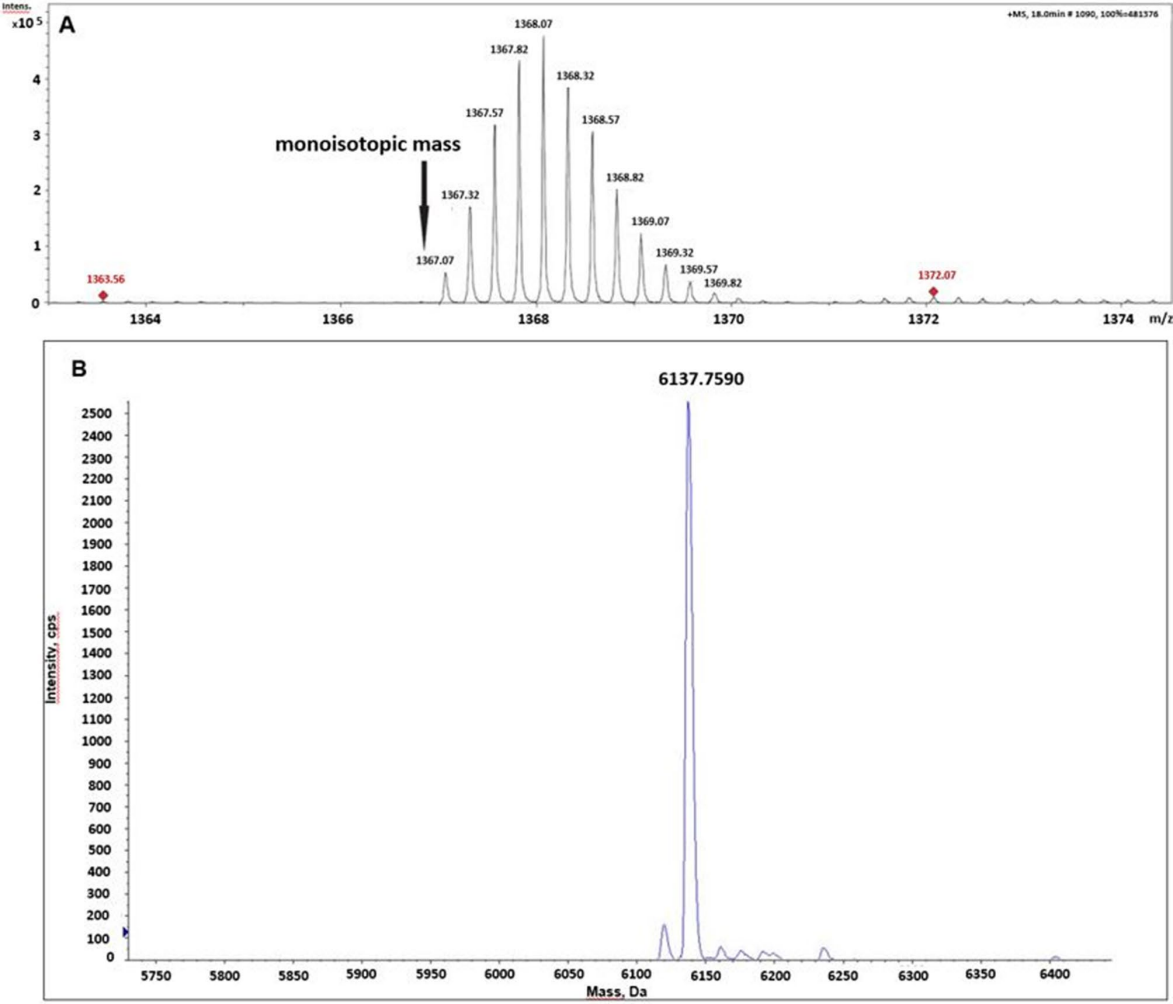
PDFL1.1 and PDFL2.1 contain the disulfide bridges. (**A**) ESI–MS spectrum of PDFL1.1 shows the mass of 5468.28 Da which corresponds to the calculated mass of 5468.22 Da (-6 H) confirming that the peptide has 3 disulfide bridges. (**B**) TOF–MS spectrum of PDFL2.1 shows the mass of the peptide as 6137.75 Da confirming the calculated mass of 6138.22 Da (-6 H).

As mentioned before, the sequence of all PDFL peptides included both the CSαβ motif^15^ and the γ-core^4^. Both motifs are frequently found in antimicrobial peptides, indicating that PDFL peptides might also have antimicrobial activity. In order to test for antimicrobial activity, it was necessary to produce the peptides in a heterologous expression system because the low expression in planta excluded the possibility to isolate the peptides from Arabidopsis plants in the amounts needed for in vitro tests. We fused the coding sequences for PDFL1.1 (with a pI of 8.6) and PDFL2.1 (with a pI of 7.6) to a 6xHis-thioredoxin tag separated by a TEV site contained in the vector pETtrx_1a. Expression in the *E.coli* C3030 strain was induced by IPTG and the fusion protein was isolated by affinity chromatography. Fusion proteins were digested with TEV protease and the PDFL peptides were purified by reversed phase chromatography (Figs. 6, 7). Correct formation of the disulfide bridges was confirmed by ESI–MS or MALDI-TOF-MS, respectively (Fig. 8).

**Figure 9:**
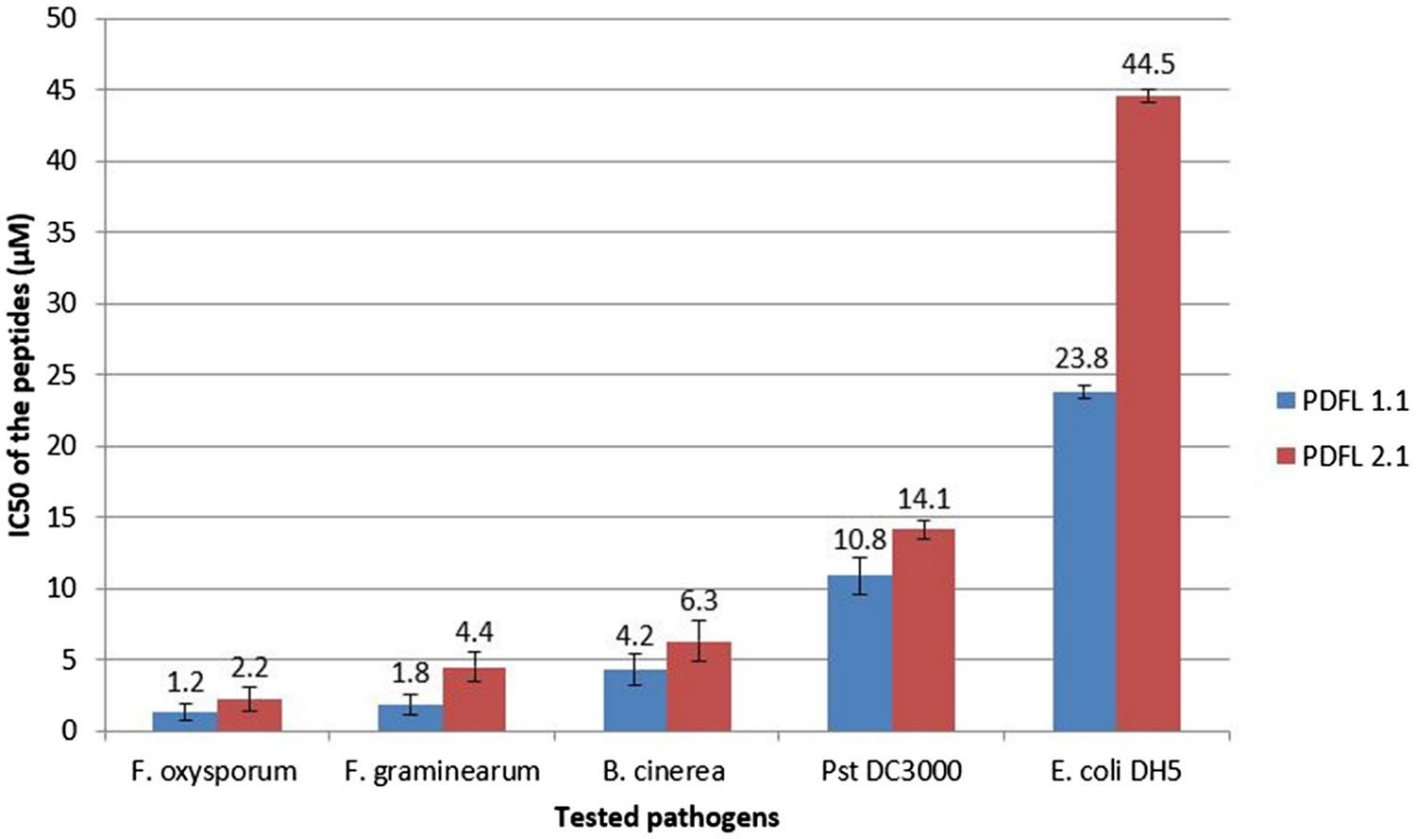
Comparison of the IC_50_ of PDFL peptides against tested pathogens. The IC50 was determined in triplicate assays as described in “Materials and methods”.

**Figure 10:**
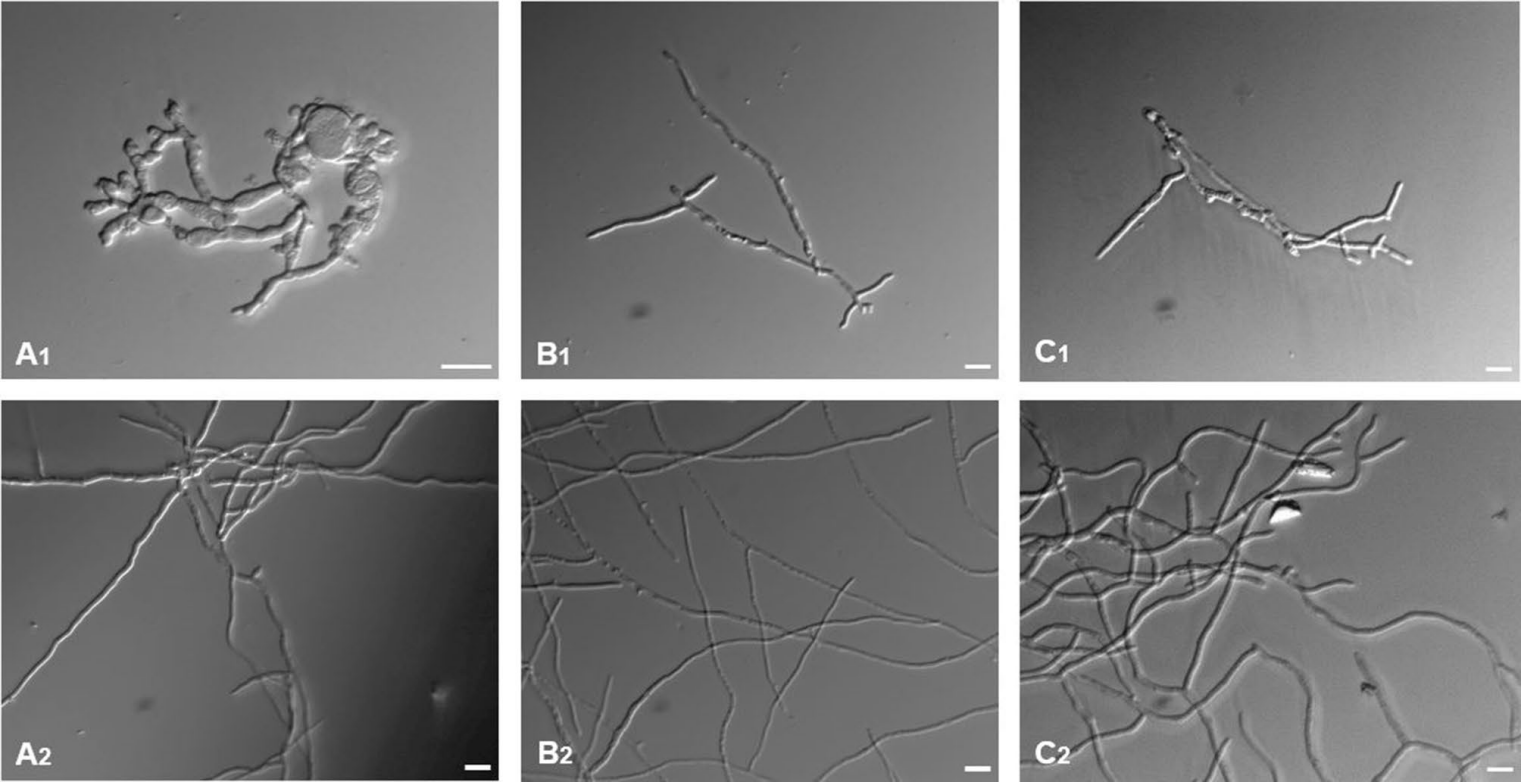
Effect of PDFL2.1 (100 µg/ml) on fungi. (**A**) A1: Hyperbranching and swelling of *Fusarium graminearum* hyphae, A2: Untreated hyphae of *F. graminearum*. (**B**): (**B1**) Inhibition of the hyphal growth of *F. oxysporum* f.sp. *matthiolae* without inducing hyperbranching, (**B2**) Untreated hyphae of *F. oxysporum* f.sp. *matthiolae*. (**C**): (**C1**) Inhibition of the hyphal growth of *Botrytis cinerea* without inducing hyperbranching, (**C2**) untreated hyphae of *B. cinerea*. Scale bar = 20 µm.

Antimicrobial activity was tested against the bacteria *E. coli* DH5alpha and *Pst* DC3000 and the fungi *Fusarium oxysporum* f.sp. *matthiolae*, *F. graminearum*, and *Botrytis cinerea* (Figs. 9, 10). Both peptides were more active against the fungi tested and there the strongest activity was found against *F. oxysporum* with an IC_50_ of 1.3 and 2.2 µM for PDFL1.1 and PDFL2.1, respectively. In general, PDFL1.1 had a stronger antimicrobial activity than PDFL2.1. At higher concentrations hyperbranching and swollen tips were observed in *F. graminearum* (Fig. 10) but not the other fungi that we tested.

### Transient expression of *PdfL* genes in *Nicotiana benthamiana*

**Figure 11:**
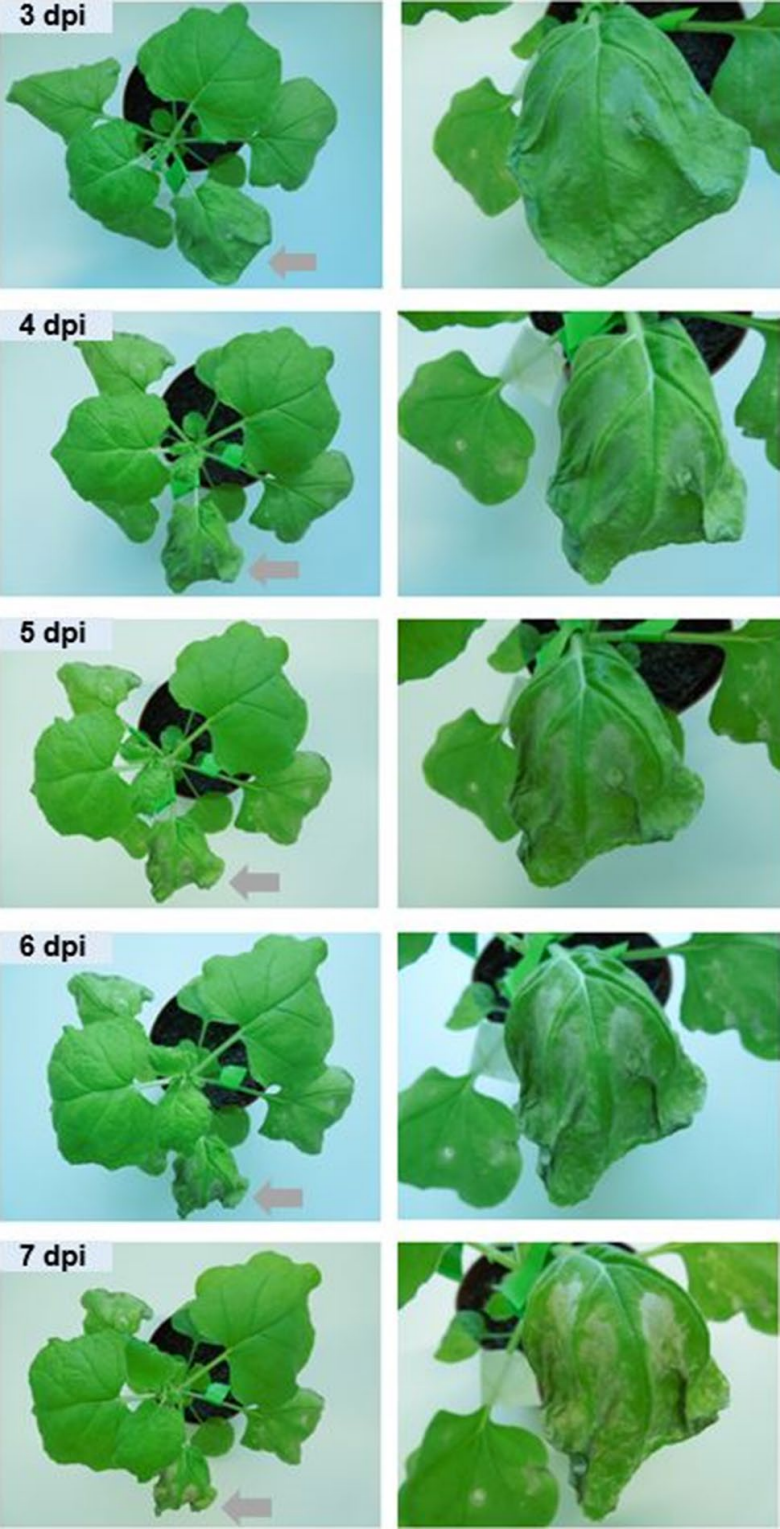
Transient expression of PDFL1.4. *N. benthamiana* leaves infiltrated with *PdfL1.4* showed strong HR at 3 dpi resulting in complete death of the infiltrated tissue at 7 dpi. The leaves indicated with gray arrows in the left side pictures are shown with magnification in the right side pictures.

**Figure 12:**
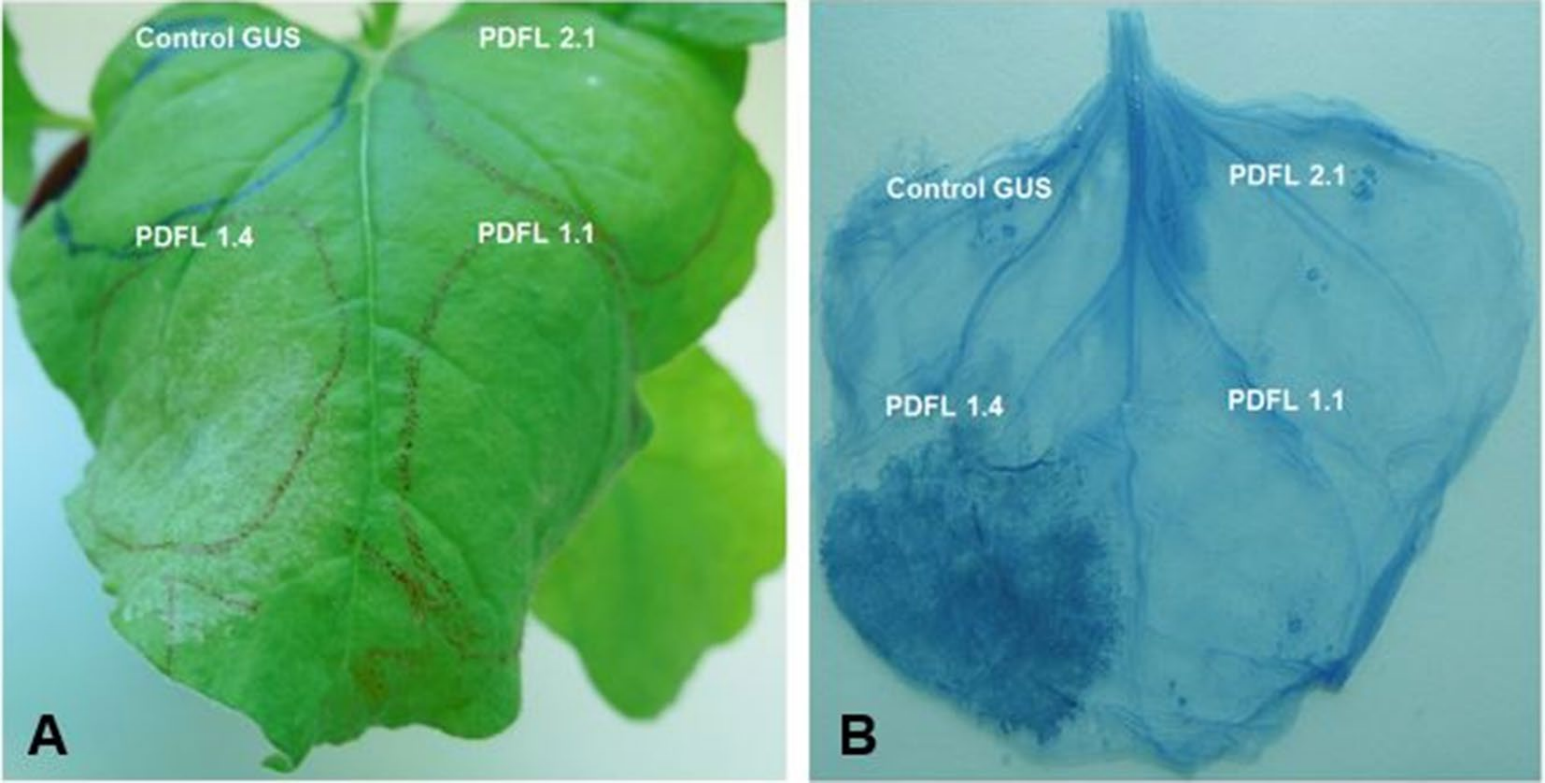
Transient expression of different PDFLs. Transient expression of PDFL 1.4 in *N. benthamiana* leaves results in strong HR in comparison to PDFL 2.1, PDFL 1.1 and control GUS. (**A**) HR starts to appear in the region that infiltrated with the PDFL 1.4 construct at 60 h after infiltration. (**B**) Cell-death is visualized with trypan blue staining.

We cloned the coding sequences of all *PdfL* genes in the plant expression vector pPZP3425. All constructs were transformed in the Agrobacterium strain GV3101. These were infiltrated in *N. benthamiana* leaves together with the RNAi silencing inhibitor P19. We observed a strong HR in case of *PdfL1.4* (Fig. 11). Cell death was confirmed by trypan blue staining (Fig. 12). Several small necrotic lesions were observed for *PdfL1.1*, *PdfL2.2*, *PdfL3.1*, *PdfL3.2*, and *PdfL4.1* (Figure S15). In case of *PdfL1.2*, *PdfL1.3*, and *PdfL2.1* only sporadic very small lesions were observed (Figure S16).

### Enhanced resistance to *F. oxysporum* in *PdfL* overexpression lines

**Figure 13:**
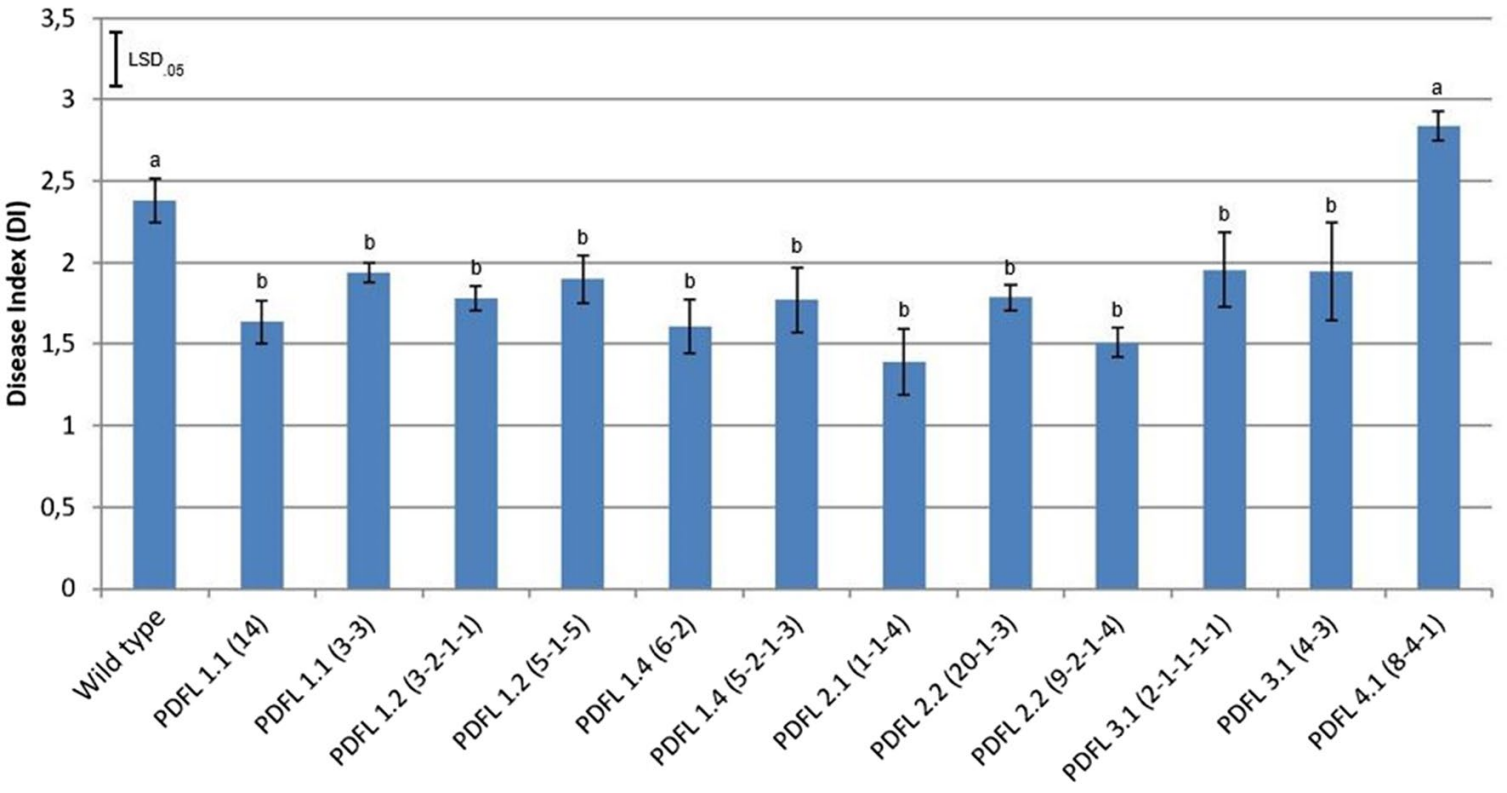
Disease index of *PdfL*s overexpression lines infected with *F. oxysporum* f. sp. *matthiolae.* For each line 4 Petri dishes were infected. Forty cotyledons from each Petri dish were evaluated for calculation of disease index and the mean value from 4 Petri dishes for each line is shown in this graph. Mean data was subjected to analysis of variance (ANOVA). The bars indicate standard error of the mean; the vertical bar represents LSD (p = 0.05) for comparing the mean values. Positive significant differences than wild-type are indicated with a different letter (b) above the columns.

The antimicrobial activity of PDFL1.1 and PDFL2.1 led us to study the function of these and other *PdfL* genes with overexpression lines. We used the vector PZP3425 and produced homozygous lines. After initial RT-PCR we tested the best lines with qRT-PCR (Figure S17). The lines which had a good expression level were then infected with *F. oxysporum*. We found that those *PdfL* overexpression lines with the exception of the *PdfL4.1* overexpression line were significantly (data was subjected to analysis of variance (ANOVA)) more resistant as compared to the wild type (Fig. 13).

## Discussion

Plant defensins are a group of antimicrobial peptides which are probably found in all plants (reviewed by Vriens et al.^17^). While the amino acid sequence is highly variable, their 3D structure resembles that of other defensins found in animals, including humans, and fungi^78^. Arabidopsis contains 13 plant defensin genes and in addition 304 *DEFL* genes^42^ which were divided into CRP groups. Here we have analysed the 9 genes which make up the CRP0240 group. We call these genes plant defensin-like -*PdfL*.

### Structure

We have recently reported the structure of PDFL2.1^75^. As other defensins, it consists of one α-helix and one triple-stranded antiparallel β-sheet. The structure is stabilized by three disulfide bridges and a cluster of hydrophobic residues within its core. The L3 loop which is located between the β-strands 2 and 3 within the γ-core was found to be more flexible. The amino acid sequence of the L3 loop is highly variable between the PDFL peptides and also other plant defensins and could be responsible for different antifungal and receptor binding activities.

PDFL2.1 is the only PDFL peptide for which the structure has been determined experimentally. For PDFL2.2, a very similar structure is predicted by PHYRE. The PDFL peptides of group 2 might be the only ones with the typical plant defensin structure. For the majority of the other PDFL peptides a structure consisting of one α-helix and two β-sheets was predicted. However, the predictions for the other PDFL peptides except PDFL2.2 have to be treated with caution and would have to be determined experimentally.

### Antimicrobial activity

The sequences of the PDFL peptides contain 6 cysteine residues, the CSαβ motif^15^ and the γ-core^4^. These motifs are typical for antimicrobial peptides and we therefore tested the antimicrobial activity for 2 of the peptides in vitro. Plant defensins have reportedly been shown to have antimicrobial activity against fungi (reviewed by Cools et al.^79^). Antibacterial activity on the other hand is rare (reviewed by Sathoff and Samac ^80^).

In line with these reports, we found strong antimicrobial activity in vitro against the fungi that we tested which was strongest against *F. oxysporum*. Sels et al.^33^ have reported IC_50_ values for two different Arabidopsis plant defensins, PDF1.1 and PDF1.3. The values that they found (µg/ml) were for *B. cinerea* 5 and 15, for *F. graminearum* 3 and 8 and for *F. oxysporum* 5 and 25, respectively. The fungal strains that they used were different than those that we used. Therefore, a comparison has to be treated with caution. Keeping this in mind, it seems that the tested PDF peptides were more active against *B. cinerea* and *F. graminearum*. In case of *F. oxysporum,* PDF1.1 had the same activity as PDFL1.1 while PDFL2.1 was approximately twice as active as PDF1.3. In case of *F. graminearum*, but not the other 2 fungi, hyphae showed swelling and hyperbranching in response to both peptides. Such a hyperbranching response of fungal growth has also been reported for fungicides^81,82^. Also plant defensins^83,84^ and thionins from radish^85^ induced hyperbranching in fungal hyphae in vitro. For the Arabidopsis thionin THI2.1 hyperbranching in *F. oxysporum* was also observed on plants overexpressing the peptide^39^. We also found activity against *P. syringae* and low activity against *E. coli*. For all tested microorganisms PDFL1.1 showed stronger activity than PDFL2.1. This higher activity could be due to the higher pI (8.62) compared to that for PDFL2.1 (7.63). In case of PDF1.1 and PDF1.3^33^ the higher antimicrobial activity of PDF1.1 might also be due to the higher pI of PDF1.1 (10) compared to PDF1.3 (9.6). According to the PHYRE models PDFL1.1 would have one α-helix and two β-sheets while PDFL2.1 has one α-helix and three β-sheets. We do not know if this difference might have an influence on the antimicrobial activity. At the moment there are not many defensin-like peptides with six cysteine residues from plants known^86^ and, to our knowledge, IC_50_ values for these peptides have not been reported. While most plant defensins contain eight cysteine residues, insect defensins have usually six cysteines and have strong antimicrobial activity (reviewed by Koehbach^87^). This might indicate that the number of cysteine residues and the corresponding number of disulphide bridges does not define the strength of the antimicrobial activity.

In case of antifungal activity, plant defensins are known to target a variety of intracellular targets, such as interaction with nucleic acids and inhibition of ion channels, among others (reviewed by Parisi et al.^88^). Antibacterial activity of plant defensins has been less studied but it was recently reported that 2 *Medicago truncatula* defensins had different mode of action against *Pseudomonas* species. MtDef4 damaged the outer membrane while Mtdef5 seemed to inhibit translation^89^. At the moment we do not know which mechanism is used by the two DEFL peptides to exert their antimicrobial activity. Further studies are needed to reveal the mode of action of these two PDFLs and perhaps other peptides of this group.

### Expression

Analysis of the expression of the *PdfL* genes with RT-PCR found only a low expression in the organs that we analysed. An exception was the expression in flowers and siliques for some of the genes. A custom microarray^77^ confirmed these data. Promoter::GUS lines showed clear GUS expression in almost all plant parts for all *PdfL* genes. This was in contrast to the expression at the mRNA level. An explanation for this difference must consider that the GUS enzyme would accumulate over time. The mRNAs for the *PdfL* genes seem to be very short lived leading to only low steady state levels of the mRNAs. Transient expression in *N. benthamiana* resulted in small HR spots and in case of *PdfL1.4* even total death of the infiltrated tissue. Thus, the expression of the PDFL peptides seems to be detrimental to the plant. This could also explain why the expression levels in the Arabidopsis overexpression lines were not very high. Contrary to the expression in *N. benthamiana*, we could generate overexpression lines for *PdfL1.4*. However, we found that the expression resulted in a phenotype with narrow leaves that were clearly different from the wild type. At the moment we have no explanation for the different behaviour of the overexpression of this gene in *N. benthamiana* and Arabidopsis.

### Function

Generally, many plant defensins have been found to have antimicrobial activity in vitro, indicating a biological function in plant defense against pathogens (reviewed by Lacerda et al.^90^). This is supported by many reports describing increased resistance of plants overexpressing plant defensins (reviewed by Sher Khan et al.^91^). We also found that overexpression of the 2 PDFL peptides for which we demonstrated antimicrobial activity and several others resulted in enhanced resistance against *F. oxysporum*. An exception was PDFL4.1 for which the overexpression line was not significantly different in resistance compared to the wild type.

We found a developmental effect for the *PdfL1.4* overexpression lines which had smaller rosettes and clearly narrower leaves compared to the wild type (Figure S18). However, this has to be tested further, for instance with a mutant line to see if that would also have a developmental effect. The transient expression in *N. benthamiana* on the other hand gave a strong HR. The expression of other PDFL peptides in *N. benthamiana* also resulted in HR lesions although not as strong as in the case of PDFL1.4. At the moment it is not clear what these data mean for a possible function in plant defense. One might speculate that the low expression of PDFL peptides could be locally increased in HR responses to pathogen infection, especially biotrophic pathogens. In line with this it has been shown that group 1 plant defensin genes are induced in the non-host response of Arabidopsis infected with the barley powdery mildew fungus^32^.

In addition to a function in plant defense a variety of other functions have been described for plant defensins. Arabidopsis PDF1.1 does not have direct antimicrobial activity but exerts its defense mechanism by sequestering iron which is needed by microbial pathogens^34,35^. Similarly, Arabidopsis PDF2.5 and PDF2.6 have been reported to chelate cadmium^36,37^. Other plant defensins have developmental effects on plants, resulting, for instance, in inhibition of root growth^92^. LUREs are DEFL peptides from *Torenia fournieri* which act as pollen tube attractants^93^. Maize DEFL genes *ZmES1-4* are specifically expressed in the mature maize embryo sac and can directly induce pollen tube burst^94^. It might thus be possible that some of the PDFL peptides also have a function in plant development.

The *PdfL4.1* gene is, to our knowledge, the only gene of this group for which a function has been described in the literature. It was found in a screen for Arabidopsis gametophyte development mutants and has been proposed to be involved in embryo sac development^95^. In another screen the mutant was also found to be more tolerant to heat stress but more sensitive to oxidative and osmotic stress^96^. How and if these different responses might be linked to embryo sac development is currently unknown. Given that we did not find enhanced resistance of the *PdfL4.1* overexpression line against *F. oxysporum*, it is possible that this gene is not involved in plant defense against pathogens. On the other hand, the PDFL4.1 peptide has the highest pI of all PDFL peptides and could have antimicrobial activity and should be tested.

### Evolution

As has been reported in results, the original classification of some of the *PdfL* genes as orphans cannot be retained as more genome sequences have become available. The PLAZA HOM04D013081 group includes the *PdfL4.1* gene plus one gene from each plant species that contain *PdfL* homologues with the exception of *T. hassleriana*. The only gene reported from this plant species is more closely related to the *PdfL2* subfamily. This might indicate that the *PdfL4.1* homologues have an important function, perhaps in embryo sac development as has been reported for the Arabidopsis *PdfL4.1* gene^95^. *T. parvula* and *G. raimondii* have no additional *PdfL* genes. Only in the *Brassicaceae* we find an expansion of this gene family with the maximum number of 9 genes in Arabidopsis. Given the very low expression levels of these genes at the RNA level, it is not clear if the majority of these genes has an important function in the plant. Evolution could have created a reservoir of genes which could at some point acquire a function. For that reason, it would perhaps be necessary that the encoded peptides be expressed at a higher level. That this might be possible is shown by our GUS analysis of the promoters which showed the promoters in principle have a strong activity in most tissues.

## Conclusions

We have identified a family of plant-defensin related genes in Arabidopsis. For 2 of the encoded peptides, we have shown antimicrobial activity in vitro which can most likely be extended to most PDFL peptides. All genes are expressed at a very low level and might thus be a reservoir of possible defense genes.

## Supplementary Information

Supplementary Information.

## Data Availability

Material is available from the first author.

## Abbreviations

AMP: Antimicrobial peptide
DEFL: Defensin-like
PdfL: Plant defensin-like
CRP: Cysteine rich peptide
MW: Molecular weight
pI: Isoelectric point
TEV: Tobacco etch virus
MS: Murashige and Skoog
*Pst* DC3000: *Pseudomonas syringae* Pv *tomato* DC3000
LSD: Least significant difference
IPTG: Isopropyl β-d-1-thiogalactopyranoside
HR: Hypersensitive response
