# Supplementary material for: Analysis of a gene family for PDF-like peptides from Arabidopsis
Source: Sci Rep. 2021 Sep 23;11:18948. doi: 10.1038/s41598-021-98175-6 (PMC8460643; doi:10.1038/s41598-021-98175-6)
Supplement: Supplementary file 1 — Supplementary Information. [file 41598_2021_98175_MOESM1_ESM.docx]

**Supplementary Information**

**Analysis of a Gene Family for PDF-Like Peptides from Arabidopsis**

Reza Omidvar^1,2^, Nadine Vosseler^1^, Amjad Abbas^1,3^, Birgit Gutmann^1,4^, Clemens Grünwald-Gruber^5^, Friedrich Altmann^5^, Shahid Siddique^1,6^, and Holger Bohlmann^1^

**Table S1:** *E. coli* strains and plasmids/vectors used in this study

| *E. coli* strain / plasmid | Purpose |
| --- | --- |
| DH10B | cloning strain |
| SHuffle C3030 | protein expression strain |
| Rosetta (DE3)pLysS | TEV_SH_ Protease production |
| DH5alpha | Activity tests |
| pTH24:TEV_SH_ | expression plasmid for TEV |
| pETtrx_1a | PDFL1.1 and PDFL2.1 expression as fusion in *E. coli* |
| pPZP3425 | plant transformation |

**Table S2:** PCR primer pairs used for creation of promoter::GUS fusions and size of product. Restriction sites are underlined.

| **Gene** | **Primer pairs*** | **Primer sequence (5´** **> 3´)** | **Product size (bp)** |
| --- | --- | --- | --- |
| *At1g64195*  *PdfL1.1* | GUS08forEcoRI | ATCGGAATTCGCTCTTCGGCAA | 721 |
|  | GUS08revNcoI | TAGACGCCATGGTTCTTATTGAAG |  |
| *At1g69818*  *PdfL1.2* | GUS06forKpnI | GTGACATGGTACCATATTACTATCAT | 1141 |
|  | GUS06revNcoI | GTTAGACGCCATGGATCTTCTTTCTT |  |
| *At1g69825*  *PdfL1.3* | GUS05forEcoRI | ATATTTGAATTCTTAAATAATGTGGA | 1020 |
|  | GUS05revNcoI | GTTAGATGCCATGGTCTTATCTGAGTT |  |
| *At1g69828*  *PdfL1.4* | GUS07forEcoRI | ATACATGGAATTCTTCTTTTGGATAT | 565 |
|  | GUS07revNcoI | TAGATGCCATGGTCTTATTTGAAGT |  |
| *At1g35537*  *PdfL2.1* | GUS04forEcoRI | ACGTTCTGAATTCTTCTCTTACTAAGAC | 1016 |
|  | GUS04revNcoI | TGATGAAACCATGGTAATCCTTTTCTT |  |
| *At4g29033*  *PdfL2.2* | GUS03forEcoRI | ACTTGGAATTCGGAAATCTTCGGGAC | 1068 |
|  | GUS03revNcoI | GCTTGATGAAGCCATGGTGATTTAT |  |
| *At3g27831*  *PdfL3.1* | GUS02forEcoRI | GTACTGAATTCGTGGATTAATTATAT | 1076 |
|  | GUS02revNcoI | GATGAAGCCATGGTTGGTTTTTGTA |  |
| *At3g27835*  *PdfL3.2* | GUS01forEcoRI | AACCGAATTCTATGCCTTTATGAAAC | 929 |
|  | GUS01revNcoI | CCTGATGATGCCATGGTTGGTTTTGT |  |
| *At4g13235*  *PdfL4.1* | GUS09forEcoRI | TGACTTCGAATTCAAGTCCGATTC | 1040 |
|  | GUS09revNcoI | TTCACGGCCATGGTTGTCTTAGTC |  |

**Table S3:** PCR primer pairs used for creation of overexpression constructs and size of product. Restriction sites are underlined.

| **Gene** | **Primer pairs*** | **Primer sequence (5´** **> 3´)** | **Product size (bp)** |
| --- | --- | --- | --- |
| At1g64195  *PdfL1.1* | DL08forNocI | TCAATAAGAACCATGGCGTCTAACAA | 363 |
|  | DL08revBamHI | AATGGGATCCTGTTTATCCTTCATAAC |  |
| At1g69818  *PdfL1.2* | DL06forNocI | GAAAGAAGATCCATGGCGTCTAACAAAG | 504 |
|  | DL06revBamHI | GATGGATCCTTATCCTACATAGCATTC |  |
| At1g69825  *PdfL1.3* | DL05forNcoI | CAGATAAGACCATGGCATCTAACAA | 454 |
|  | DL05revBamHI | GATGGATCCTTATCCTACATAACACTC |  |
| At1g69828  *PdfL1.4* | DL07forNcoI | CAAATAAGACCATGGCATCTAACAAAG | 450 |
|  | DL07revBamHI | TAAGGATCCTTATCCTACTTTACATACACAC |  |
| At1g35537  *PdfL2.1* | DL04forNcoI | AAAAGGATTACCATGGTTTCATCAA | 386 |
|  | DL04revBamHI | CATGGATCCTTATATATAATATTTTGGGAAC |  |
| At4g29033  *PdfL2.2* | DL03forNcoI | ATAAATCACCATGGCTTCATCAAGC | 433 |
|  | DL03revBamHI | AATGGATCCCTTAAACATGTGTTATAG |  |
| At3g27831  *PdfL3.1* | DL02forNcoI | AAAACCAACCATGGCTTCATCAAGC | 634 |
|  | DL02revBamHI | TTAGGATCCAGATCATGTATGAATACA |  |
| At3g27835  *PdfL3.2* | DL01forNcoI_2 | ACAACCATGGCATCATCAGGCAAATGT | 632 |
|  | DL01revBamHI_2 | TTAGGATCCAGATTATGAATAAATACAAGTACA |  |
| At4g13235  *PdfL4.1* | DL09forNocI | AGACAACCATGGCCGTGAAGCTC | 400 |
|  | DL09revBamHI | TGATGGATCCTAAGTATATTTACAG |  |

**Table S4:** Specific primer pairs for *PdfL* genes and size of amplified products using genomic DNA or cDNA as templates.

| **Gene** | **Primer pairs*** | **Primer sequence (5´** **> 3´)** | **Product size (bp)** | |
| --- | --- | --- | --- | --- |
|  | | | **genomic** | **cDNA** |
| *At1g64195*  *PdfL1.1* | At1g64195for | CCTTTGTTCTTTGCCTATATATGTG | 298 | 184 |
|  | At1g64195rev | ACACTCGCACATGTAATTACC |  |  |
| *At1g69818*  *PdfL1.2* | At1g69818for | CTTCTTGGTTCTTTGCCTTTGTG | 406 | 145 |
|  | At1g69818rev | ACCCATATAATTTTGATTAAGG |  |  |
| *At1g69825*  *PdfL1.3* | At1g69825for | CATCTTTATTCTTTTTCTATGTG | 397 | 175 |
|  | At1g69825rev | ACTCACACATATAGTGATTGG |  |  |
| *At1g69828*  *PdfL1.4* | At1g69828for | TTTCTTGGTTCTTTGTCTATGTA | 390 | 188 |
|  | At1g69828rev | ATACACACATATAACCTTGATC |  |  |
| *At1g35537*  *PdfL2.1* | At1g35537for | TGTATGGTTGTTCTCTTGAA | 303 | 184 |
|  | At1g35537rev | AAACACATACAAAGTCAAGGC |  |  |
| *At4g29033*  *PdfL2.2* | At4g29033for | TGTGTTTGGCTGCTCTCTTG | 327 | 152 |
|  | At4g29033rev | GAAATGCATCAAATGGTATCCA |  |  |
| *At3g27831*  *PdfL3.1* | At3g27831for | CTTCATCAAGCAAATGCGCC | 568 | 190 |
|  | At3g27831rev | TTAGGTGGACAATGCCTCAA |  |  |
| *At3g27835*  *PdfL3.2* | At3g27835for | GCATCATCAGGCAAATGTGT | 525 | 199 |
|  | At3g27835rev | CCAGGTGGAACATGTCTCAAAC |  |  |
| *At4g13235*  *PdfL4.1* | At4g13235for2 | TGGCCGTGAAGCTCATTTAC | 223 | 117 |
|  | At4g13235rev2 | GCTACGTACAGCCATTCCGT |  |  |

**Table S5:** PCR primer pairs used for cloning of *PdfL1.1* and *PdfL2.1* genes in pETtrx_1a.

| **Primer** | **Primer sequence (5´→ 3´)** |
| --- | --- |
| pETtrxfor1 | GTCCGGCGTAGAGGATCG |
| pETtrxfor2 | TCCCGCGAAATTAATACGACT |
| pETtrxTEVrev | TCTGAGAATCTTTATTTTCAG |
| At1g35537fortev | CTTTATTTTCAGAAAGATATTGATGGAAGG |
| At1g35537revBamHI | CATGGATCC TTATATATAATATTTTGGGA |
| At1g64195fortev | CTTTATTTTCAGAAAAGTATGAATCCAAC |
| At1g64195revBamHI | AGAGGATCCTTATCCTTCATAACACTC |

**Table S6:** Size of PCR products amplified by primers of table S5.

| **Primer pairs** | **PCR product (bp)** |
| --- | --- |
| pETtrxfor1  pETtrxTEVrev | 515 |
| At1g35537fortev  At1g35537revBamHI | 189 |
| At1g64195fortev  At1g64195revBamHI | 174 |
| pETtrxfor2  At1g35537revBamHI | 665 |
| pETtrxfor2  At1g64195revBamHI | 650 |

**Table S7:** Microarray data from Tesfaye et al. (2013) - expression in different plant parts.

INF, inflorescence

Root, root at 21d

sedl14, seedling at 14d

sedl7, seedling at 7d

| Gene | INF | Root | sedl14 | sedl7 | siliques |
| --- | --- | --- | --- | --- | --- |
| *At1g35537* | 21.8 | 15.3 | 14.4 | 13.2 | 38.3 |
| *At1g64195* | 13.1 | 18.5 | 11.3 | 11.0 | 14.7 |
| *At1g69818* | 13.0 | 8.1 | 9.1 | 8.5 | 18.2 |
| *At1g69825* | 7.3 | 7.6 | 9.2 | 7.5 | 12.2 |
| *At1g69828* | 14.8 | 15.8 | 16.5 | 15.7 | 15.9 |
| *At3g27831* | 10.7 | 10.5 | 10.1 | 10.4 | 67.1 |
| *At3g27835* | 8.8 | 9.1 | 9.4 | 8.9 | 84.1 |
| *At4g13235* | 12.4 | 195.0 | 15.8 | 67.4 | 10.6 |
| *At4g29033* | 8.2 | 8.7 | 9.0 | 8.1 | 21.2 |

**Table S8:** Microarray data from Tesfaye et al. (2013) - expression in leaves after infection with *Alternaria brassicicola*.

ALTMOCK, Mock-inoculated Columbia leaf - control for *A. brassicicola*

ALTCO24, *A. brassicicola* _inoculated Columbia

ALTDDE224, *A. brassicicola* _inoculated mutant *dde2*;

| Gene | ALTMOCK | ALTCO24 | ALTDDE224 |
| --- | --- | --- | --- |
| *At1g35537* | 14.7 | 17.0 | 33.3 |
| *At1g64195* | 12.4 | 23.4 | 11.9 |
| *At1g69818* | 7.5 | 6.9 | 6.7 |
| *At1g69825* | 6.6 | 6.3 | 6.1 |
| *At1g69828* | 17.9 | 18.8 | 34.3 |
| *At3g27831* | 9.7 | 8.7 | 9.7 |
| *At3g27835* | 7.4 | 7.9 | 7.3 |
| *At4g13235* | 8.2 | 6.9 | 6.1 |
| *At4g29033* | 7.8 | 7.7 | 6.8 |

**Table S9:** Microarray data from Tesfaye et al. (2013) - expression in leaves after infection with *Pseudomonas syringae* pv *tomato*.

PST3MOCK, Mock-inoculated control leaf for *P. syringae* - at 3 h

PST9MOCK, Mock-inoculated control leaf for *P. syringae* - at 9 h

AVR3, AvrRpt2-inoculated leaf at 3h

AVR9, AvrRpt2-inoculated leaf at 9h

DC3003, DC3000-inoculated leaf at 3h

DC3009, DC3000-inoculated leaf at 9h

Hrcc3, Hrcc-inoculated leaf at 3h

Hrcc9, Hrcc-inoculated leaf at 9h

(PtoDC3000hrcC is a strain unable to deliver effectors which induce PTI. PtoDC3000AvrRpt2 delivers AvrRpt2, an effector that triggers ETI in Col-0 strains compared to plants inoculated with wild type PtoDC3000.)

| Gene | PST3MOCK | PST9MOCK | AVR3 | AVR9 | DC3003 | DC3009 | Hrcc3 | Hrcc9 |
| --- | --- | --- | --- | --- | --- | --- | --- | --- |
| *At1g35537* | 14.5 | 12.0 | 15.3 | 17.9 | 13.9 | 12.3 | 13.3 | 14.0 |
| *At1g64195* | 11.4 | 9.1 | 10.8 | 14.0 | 11.6 | 13.7 | 10.4 | 11.4 |
| *At1g69818* | 8.2 | 6.3 | 6.8 | 7.4 | 7.4 | 5.5 | 7.4 | 6.9 |
| *At1g69825* | 6.8 | 5.7 | 6.9 | 6.9 | 7.5 | 4.2 | 7.2 | 6.4 |
| *At1g69828* | 16.6 | 13.9 | 18.7 | 22.0 | 15.2 | 16.5 | 14.7 | 16.8 |
| *At3g27831* | 9.3 | 9.6 | 8.8 | 10.5 | 8.6 | 9.3 | 9.3 | 10.1 |
| *At3g27835* | 7.9 | 7.5 | 8.1 | 7.4 | 7.8 | 6.3 | 8.6 | 8.2 |
| *At4g13235* | 9.9 | 6.9 | 7.5 | 8.9 | 9.4 | 7.2 | 9.4 | 8.9 |
| *At4g29033* | 8.8 | 9.0 | 8.4 | 10.4 | 8.2 | 6.6 | 8.3 | 8.5 |

**Figure S1:** Representative trypan blue staining of four different classes of the fungal hyphae growth on cotyledons. (A) No visible infection (class 0). (B) 1-20 hyphae (Class 1). White arrows indicate the hyphae. (C) 20-100 hyphae (class 2). (D) Densely covered cotyledon (class 3). Bar = 100 µm.


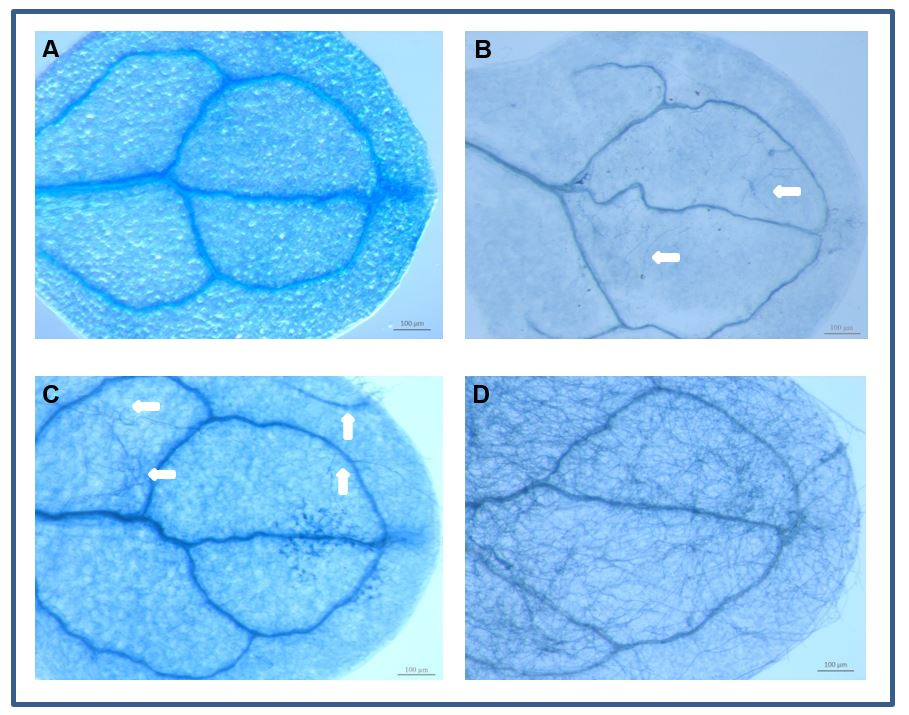


**Figure S2**: Homologous *PdfL* genes


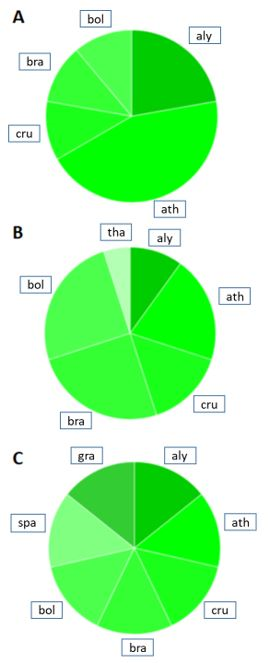


Homologous genes in the PLAZA gene families HOM04D011286 (A), HOM04D008815 (B), and HOM04D013081 (C). ath, *A. thaliana*; aly, *A. lyrata*; bol, *Brassica oleracea*; bra, *B.* *rapa*; cru, *Capsella rubella*; spa, *Schrenkiella* (*Thellungiella*) *parvula*; tha, *Tarenaya* *hassleriana*; gra, *Gossypium raimondii*.

**Figure S3**: Phyre 3D structure prediction (<http://www.sbg.bio.ic.ac.uk/~phyre2/html/>)


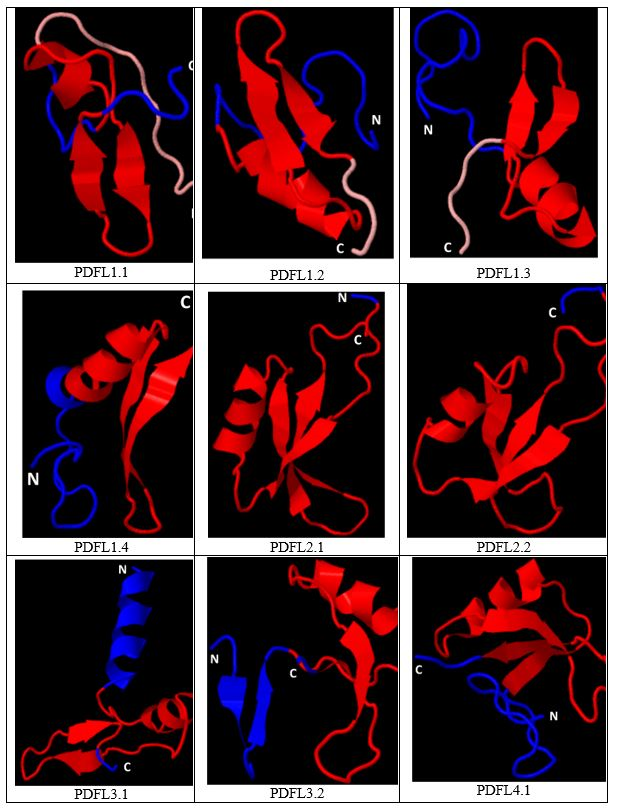


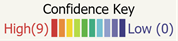


**Templates that were used for the models**


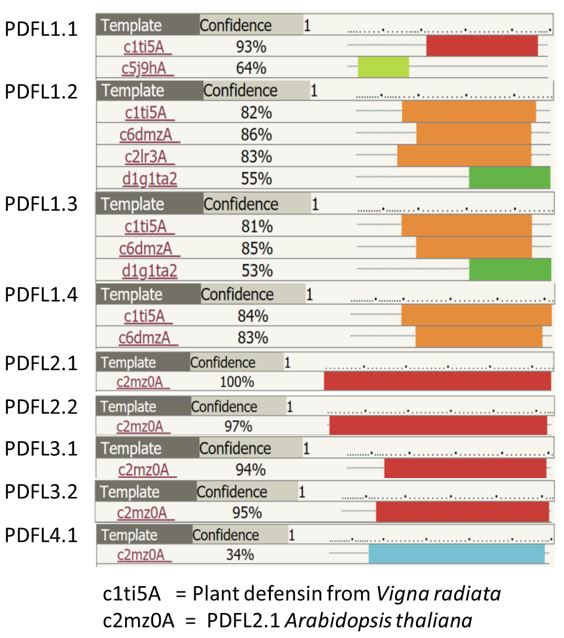


**Figure S4**


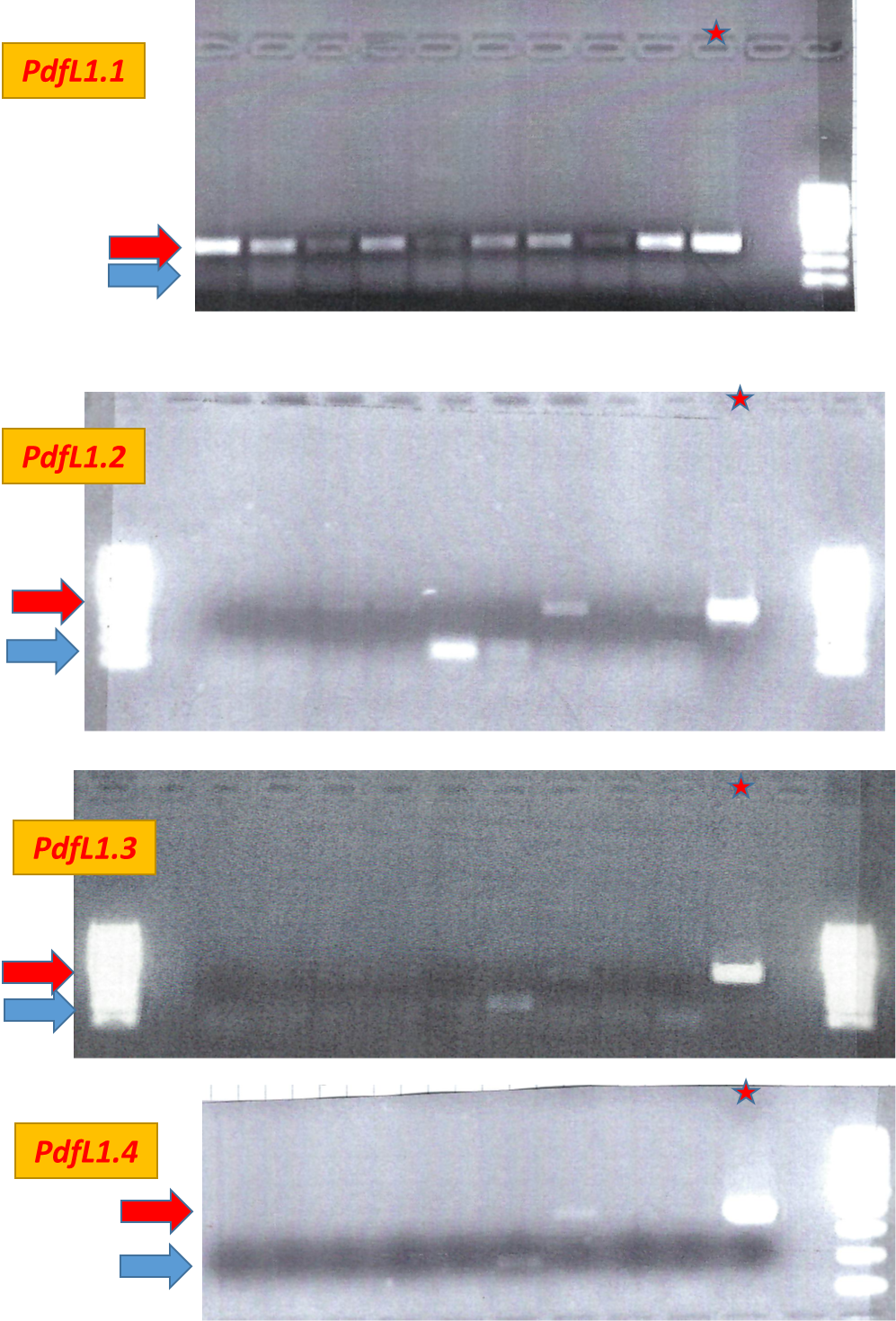


**
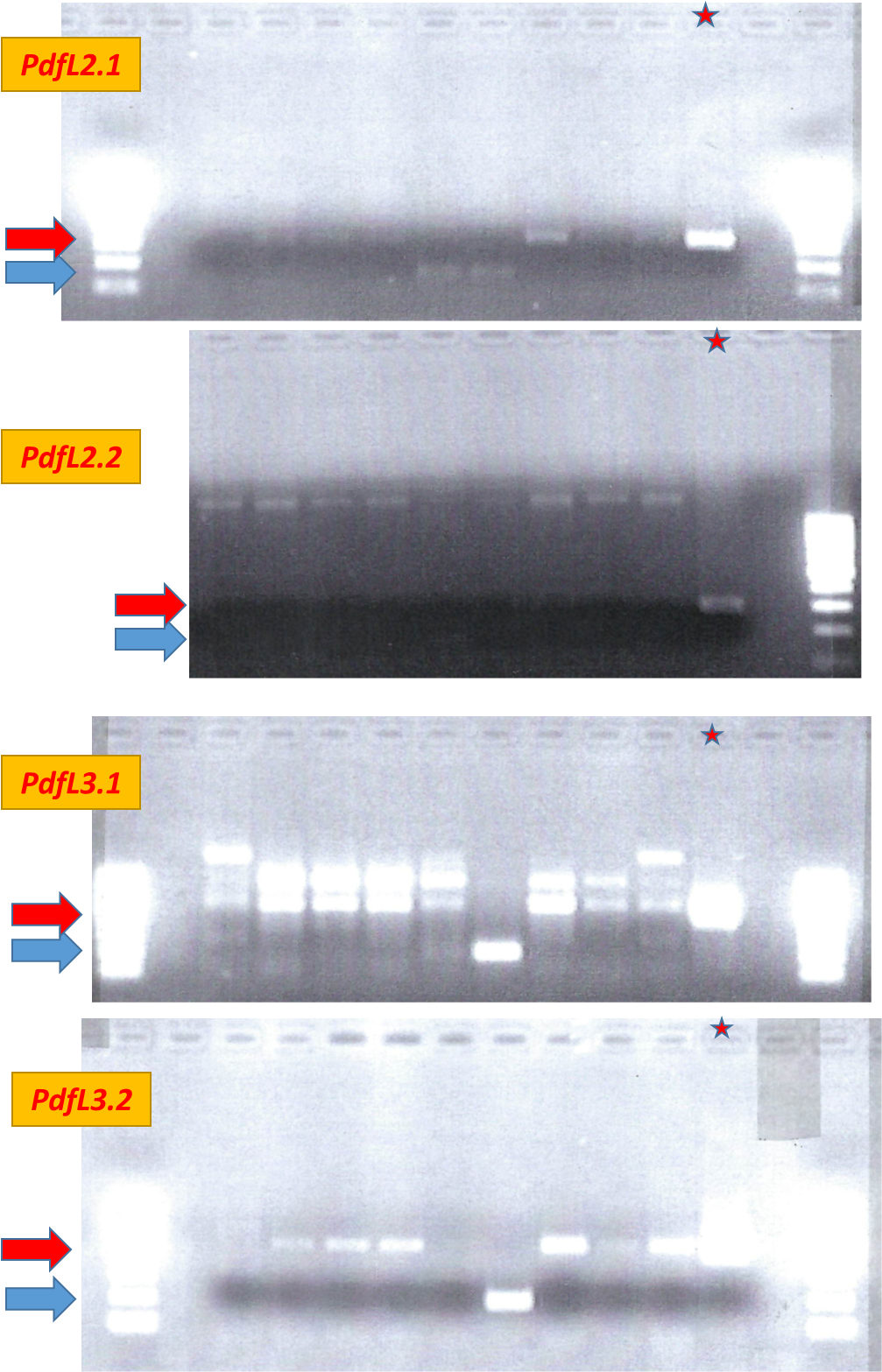
**

**
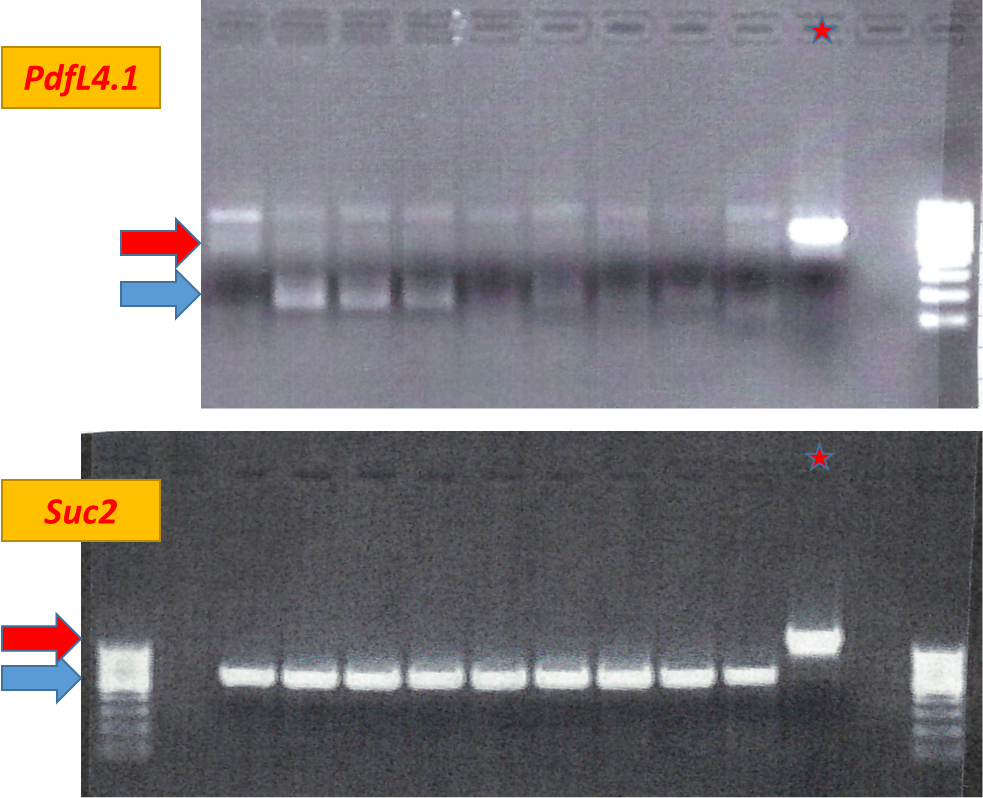
**

RT-PCR for *PdfL* genes in different Arabidopsis tissues.

Red star shows the lane with amplified genomic DNA control. Probes from left to right on the left side of genomic DNA: roots 14 days on MS medium; seedlings 5 days MS; rosette leaves 5 weeks; seedlings 14 days MS; flowers; siliques; stems; cauline leaves; roots 5 days MS; genomic DNA. Total RNA was transcribed using oligo-dT and superscriptIII reverse transcriptase and amplified using gene-specific primers placed on both sides of the intron. Blue arrows show the size of the cDNA fragment while red arrows show the size of amplified genomic DNA which is always larger because of the intron.


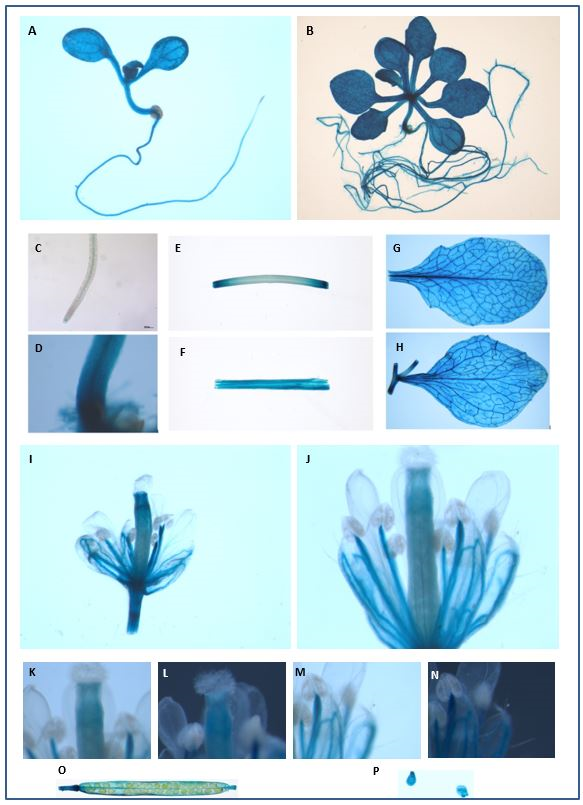


**Figure S5:** Promoter activities of *PdfL1.1* gene in different tissues at various developmental stages. (A) 5 days old seedling. (B) 14 days old seedling. (C) Root tip. (D) Hypocotyl. (E) Stem. (F) Cut stem. (G) 5 weeks old rosette leaves. (H) 5 weeks old cauline leaves. (I) Open flower. (J) Magnified view of open flower. (K) and (L) Female organ. (M) and (N) Male organ. (O) Siliques. (P) seeds. 102


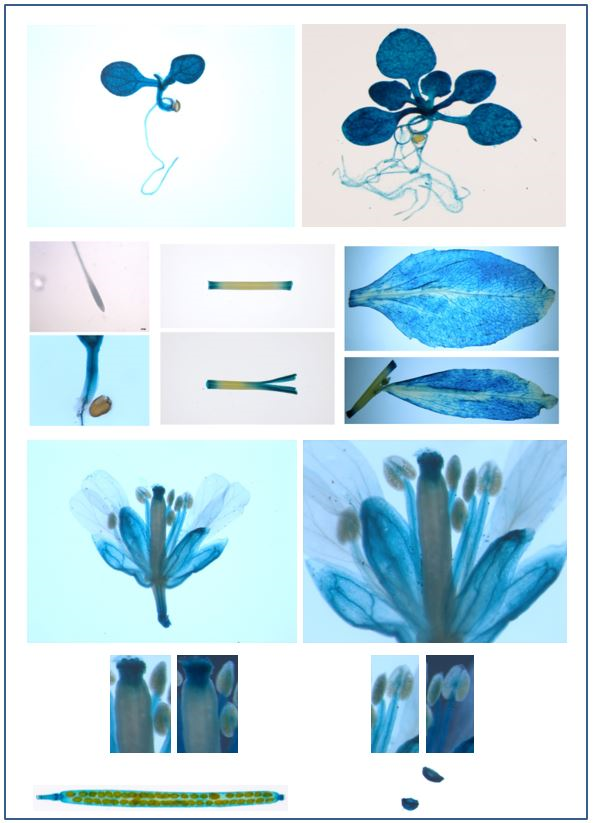


**Figure S6:** Promoter activities of *PdfL1.2* gene in different tissues at various developmental stages.


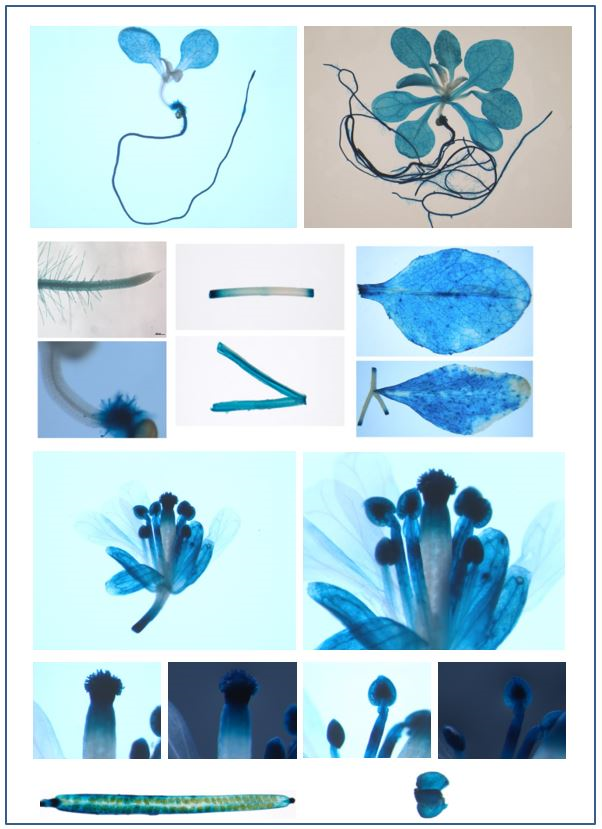


**Figure S7:** Promoter activities of *PdfL1.3* gene in different tissues at various developmental stages.


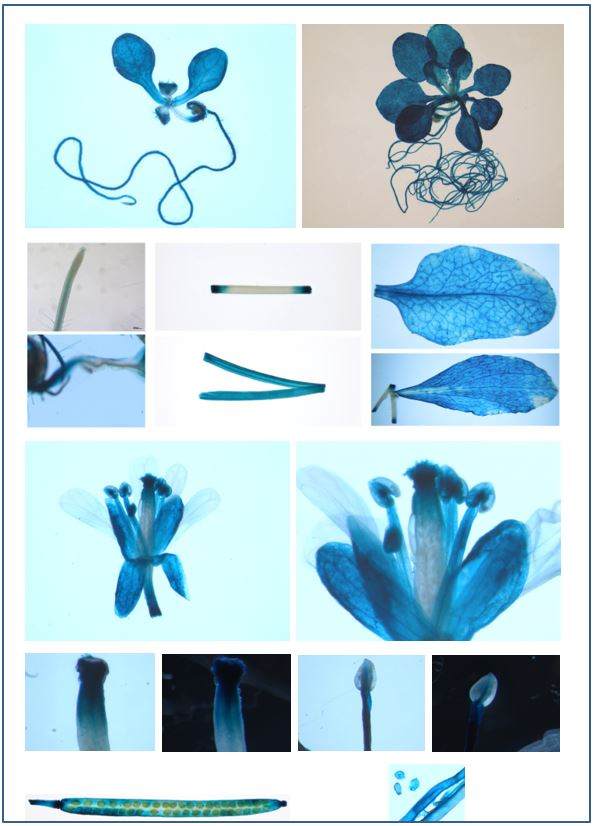


**Figure S8:** Promoter activities of *PdfL1.4* gene in different tissues at various developmental stages.


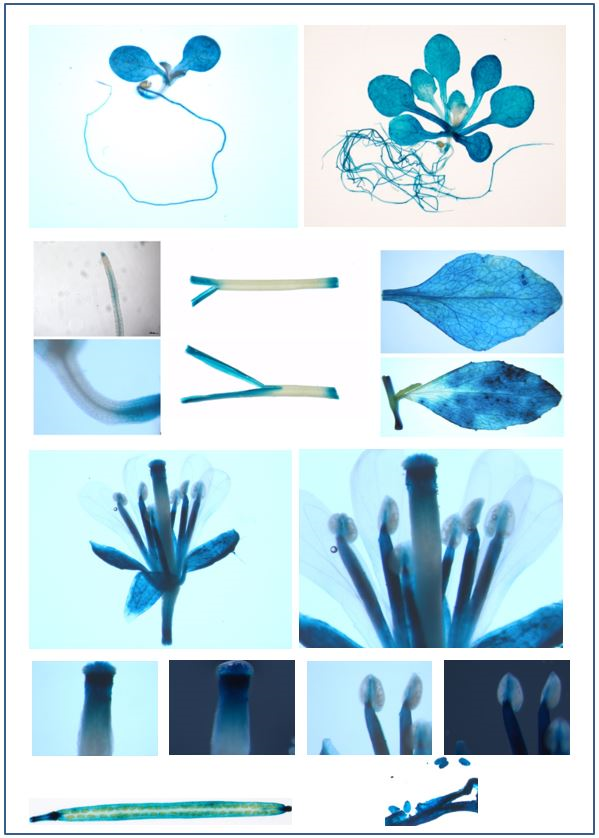


**Figure S9:** Promoter activities of *PdfL2.1* gene in different tissues at various developmental stages.


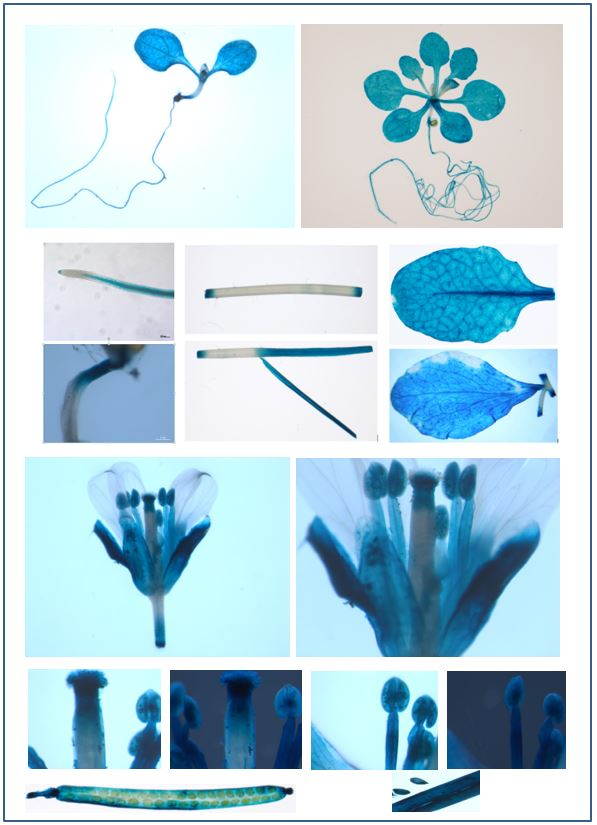


**Figure S10:** Promoter activities of *PdfL2.2* gene in different tissues at various developmental stages.


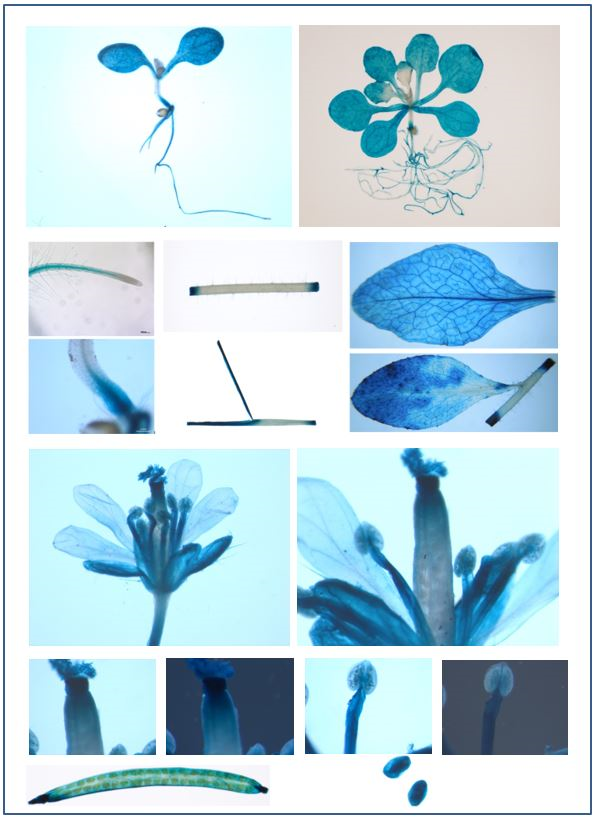


**Figure S11:** Promoter activities of *PdfL3.1* gene in different tissues at various developmental stages.


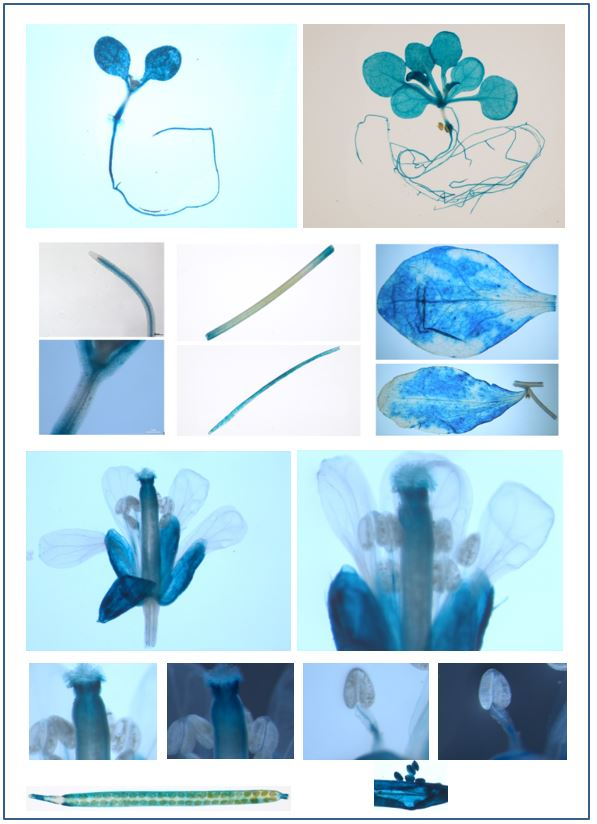


**Figure S12:** Promoter activities of *PdfL3.2* gene in different tissues at various developmental stages.


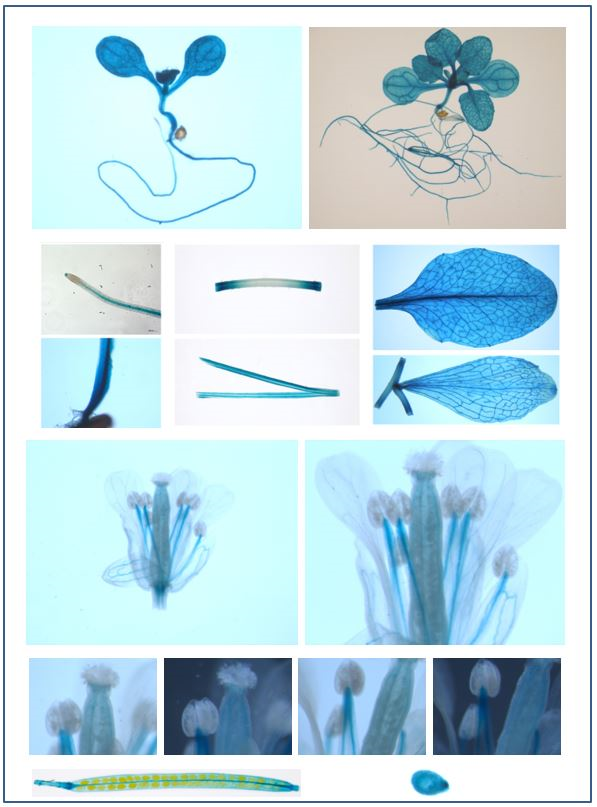


**Figure S13:** Promoter activities of *PdfL4.1* gene in different tissues at various developmental stages.

**Figure S14: Full-length gels related to Figure 7 in the manuscript.**

**(A):** **His-tag purification of fusion protein.** 1: Uninduced crude fraction, 2: Induced crude fraction, 3: Total soluble protein, 4: Insoluble fraction, 5-7: First, second and third elution after purification. M: Low range protein ladder.


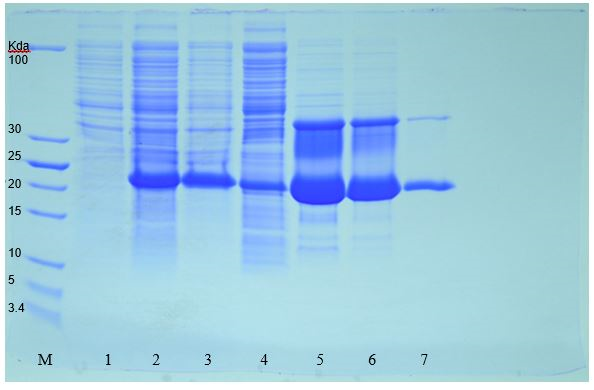


**(B): Digestion of the purified fusion protein with TEV protease.** 1: Total soluble protein, 2: Eluted protein after His-tag purification, 3: Protein after acetone precipitation and dissolving in TEV reaction buffer, 4: TEV digested fusion protein, 5: Second His-tag purification after TEV digestion, 6: Second time loading of the second His-tag purification after TEV digestion. 7-8: First and second wash after second His-tag purification with His buffer without imidiazole. 9: First elution after second His-tag purification with His buffer containing imidiazole. M: Low range protein ladder.


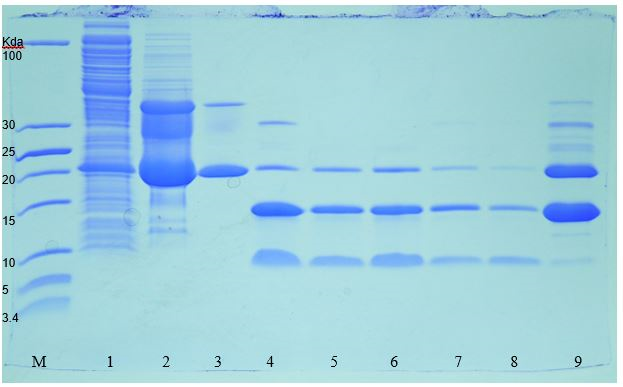


**(C): Final purification of PDFL2.1 using reverse phase chromatography.** 1: TEV digested fusion protein, 2: Purified PDFL2.1 after chromatography loaded with non-reduced sample buffer. 3-6: PDFL2.1 after HPLC loaded with reduced sample buffer (dimers are formed). 7-9: Not-related to this experiment. M: Low range protein ladder.


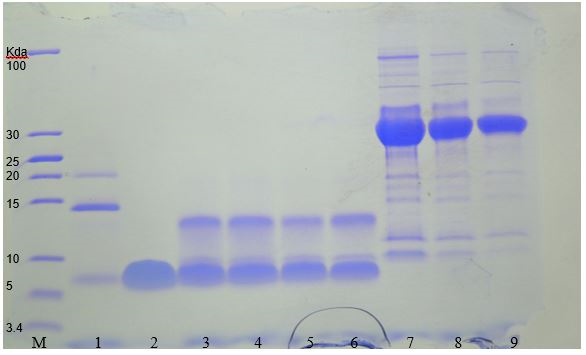


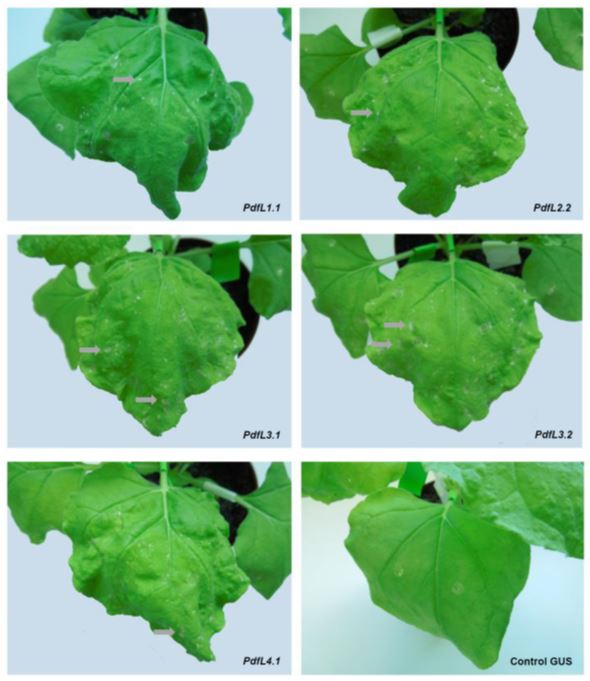


**Figure S15:** Visible small necrotic lesions appear at 3 dpi on the leaves infiltrated with *PdfL1.1*, *PdfL2.2*, *PdfL3.1*, *PdfL3.2* and *PdfL4.1* in comparison to the control GUS. Some of the necrotic lesions are indicated with gray arrows.


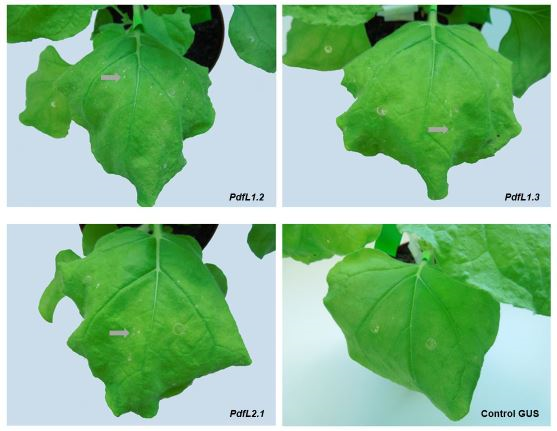


**Figure S16:** Response of infiltrated leaves to the transient expression of *PdfL1.2*, *PdfL1.3* and *PdfL2.1* in comparison to the control GUS. Only very few small necrotic lesions appear (indicated with gray arrows).


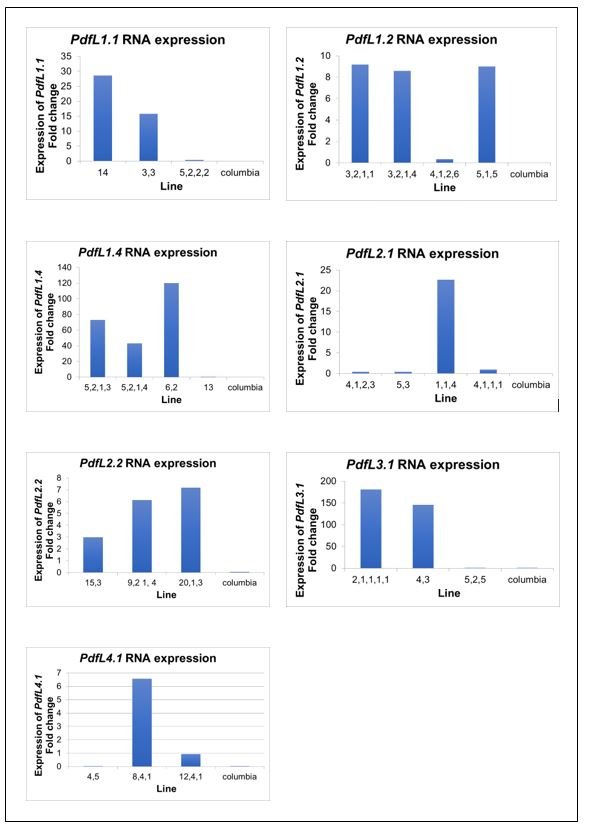


**Figure S17:** qRT-PCR of *Pdfl* overexpression lines.


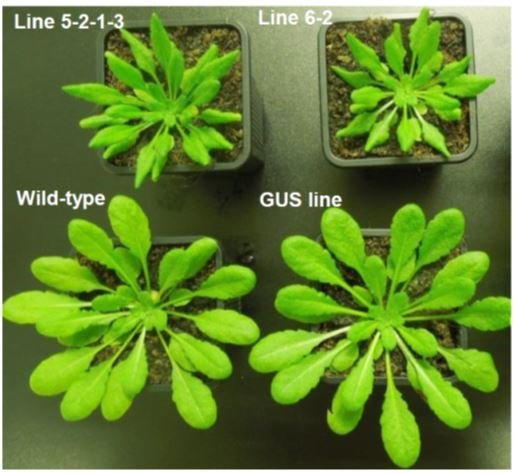


**Figure S18:** *PdfL1.4* overexpression lines 5-2-1-3 and 6-2 have narrower and smaller leaves. The smaller leaves of these lines are compared with wild-type plants and a GUS line in this picture.
